# Genome-wide identification and *in-silico* expression analysis of *CCO* gene family in sunflower (*Helianthus annuus)* against abiotic stress

**DOI:** 10.1007/s11103-024-01433-0

**Published:** 2024-04-03

**Authors:** Adnan Sami, Muhammad Zeeshan Haider, Muhammad Shafiq, Saleh Sadiq, Farooq Ahmad

**Affiliations:** 1 Department of Plant Breeding and Genetics, Faculty of Agricultural Sciences, University of the Punjab, Lahore, P.O BOX. 54590, Pakistan; 2 Department of Horticulture, Faculty of Agricultural Sciences, University of the Punjab, Lahore, P.O BOX. 54590, Pakistan; 3 Institute of Biochemistry, Biotechnology, and Bioinformatics (IBBB), The Islamia University of Bahawalpur, Bahawalpur, Pakistan; 4 Sustainable Forest Management Research Institute (iuFOR), University of Valladolid and INIA, Avenida de Madrid, Palencia, 34004 Spain; 5 Department of Vegetable Production and Forest Resources, University of Valladolid, Avda. de Madrid, Palencia, 34004 Spain

**Keywords:** Carotenoid cleavage oxygenases, RNA seq analysis, Transcription factor, *Helianthus annuus*, 9-cis-epoxycarotenoid dioxygenases, Carotenoid cleavage dioxygenases

## Abstract

Carotenoid cleavage oxygenases (CCOs) enzymes play an important role in plant growth and development by producing a wide array of apocarotenoids and their derivatives. These compounds are vital for colouring flowers and fruits and synthesizing plant hormones such as abscisic acid and strigolactones. Despite their importance, the gene family responsible for CCO enzymes in sunflowers has not been identified. In this study, we identify the *CCO* genes of the sunflower plant to fill this knowledge gap. Phylogenetic and synteny analysis indicated that the *Helianthus annuus CCO (HaCCO)* genes were conserved in different plant species and they could be divided into three subgroups based on their conserved domains. Analysis using MEME tool and multiple sequence alignment identified conserved motifs in the *HaCCO* gene sequence. *Cis*-regulatory elements (CREs) analysis of the *HaCCO* genes indicated the presence of various responsive elements related to plant hormones, development, and responses to both biotic and abiotic stresses. This implies that these genes may respond to plant hormones, developmental cues, and drought stress, offering potential applications in the development of more resistant crops. Genes belonging to the 9-cis-epoxy carotenoid dioxygenases (NCED) subgroups predominantly exhibited chloroplast localization, whereas the genes found in other groups are primarily localized in the cytoplasm. These 21 identified *HaCCOs* were regulated by 60 miRNAs, indicating the crucial role of microRNAs in gene regulation in sunflowers. Gene expression analysis under drought stress revealed significant up-regulation of *HaNCED16* and *HaNCED19*, genes that are pivotal in ABA hormone biosynthesis. During organ-specific gene expression analysis, *HaCCD12* and *HaCCD20* genes exhibit higher activity in leaves, indicating a potential role in leaf pigmentation. This study provides a foundation for future research on the regulation and functions of the *CCO* gene family in sunflower and beyond. There is potential for developing molecular markers that could be employed in breeding programs to create new sunflower lines resistant to biotic and abiotic stresses.

**Supplementary Information:**

The online version contains supplementary material available at 10.1007/s11103-024-01433-0.

## Key message

• Based on structural analyses, 21 *CCO* genes were discovered in *H. annuus* with varying number of introns (1 to 14).

• Identification of cis-regulatory elements in *HaCCO* promoters associated with light responsiveness, development and growth responsiveness, hormone regulation and abiotic stress.

• RNA-seq data identified drought stress-resistant genes (*HaNCED16* and *HaNCED19*) for potential use in developing water-limited-tolerant sunflower varieties.

• *HaCCD12* and *HaCCD20* genes showed higher activity in leaves, suggesting a role in leaf pigmentation.

• These analyses provided comprehensive genome-wide knowledge of sunflower *HaCCO* genes but further research, including gene cloning and functional analysis, is needed for confirmation.

**Supplementary Information:**

The online version contains supplementary material available at 10.1007/s11103-024-01433-0.

## Introduction

Carotenoid Cleavage Oxygenases (CCOs) are a group of enzymes that play an important role in carotenoid metabolic processing across various organisms, including plants (Haider et al. 2023). Plants, algae, and some bacteria naturally produce pigments called carotenoids, which are responsible for vibrant colors in many fruits, vegetables, and flowers. In plants, CCOs have important roles in the pigmentation of flowers and fruits, the synthesis of plant hormones like abscisic acid and strigolactones, and the regulation of physiological responses to light and stress. CCOs are comprised of two enzymes: carotenoid cleavage dioxygenases (CCDs) and 9-cis-epoxy carotenoid dioxygenases (NCED) (Poliakov et al. 2020); (Su et al. 2021). These enzymes play a crucial role in converting carotenoids into apocarotenoids, which result from the degradation of carotenoids. In this process, CCOs use oxygen to cleave the carotenoid molecule and generate apocarotenoids, which have various physiological functions, such as acting as plant hormones, attracting pollinators, and defending against environmental stress (Su et al. 2021).

NCED is a crucial enzyme in the biosynthesis pathway of abscisic acid. Its widespread investigation and identification across various plant species suggest that it holds a significant function in plant growth and development (Sami et al. 2023a). One notable aspect of NCED is its evolutionary conservation across diverse plant lineages (Rehman et al. 2022), (Sami et al. 2023b). The gene encoding NCED has been found to show a slight divergence within its subfamily, with a high degree of exon conservation. This conservation of exon position suggests the importance of NCED in maintaining the structural integrity of the protein, which is crucial for its function (Irfan et al. 2023).

Furthermore, *NCED* genes have been observed to evolve relatively slower (Song et al. 2022). This slow rate of evolution might play a role in the functional divergence of *NCED* gene subfamilies across different tissues. For example, *NCED* subfamilies may have different expression patterns and activity levels in different plant tissues, which could contribute to regulating ABA biosynthesis and plant responses to environmental cues (Nakashima et al. 2022). Therefore, identifying the *NCED* gene is critical in studying ABA biosynthesis and its function in plant growth and development. First discovered in the maize ABA-deletion mutant Vp14, the *NCED* gene provided insight into the function of *NCED* in ABA production (Mohsenzadeh Golfazani et al. 2022). Later, the *NCED* gene family has been identified and investigated in many plant species, such as Arabidopsis (Tan et al. 2003), cotton (Li et al. 2021), avocado (Chernys and Zeevaart 2000), cowpea (Iuchi et al. 2000), kiwifruit (Gan et al. 2020), and grape (Wang et al. 2019). However, the identity and function of *NCED* genes in sunflower has not been studied yet.

*Carotenoid cleavage dioxygenases* (*CCDs*) genes belong to the carotenoid cleavage subfamily and are involved in diverse functions related to plant growth and stress responses. These enzymes break down certain apocarotenoid molecules by cleaving particular double bonds in carotenoids, which have important roles in plant signaling, pigmentation, and defense against environmental stresses (Yao et al. 2022). One of the most important functions of CCD enzymes is their role in the biosynthesis of abscisic acid (ABA), a hormone that regulates seed development, dormancy, and stress responses. CCD enzymes also play a role in the degradation of carotenoid pigments, which can impact the coloration of plant tissues (Wei et al. 2016). In addition, some apocarotenoids produced by CCD enzymes have been shown to have antioxidant, antimicrobial, and other bioactive properties. Overall, CCD enzymes are important players in the complex plant signaling network and adaptation to changing environmental conditions (Yue et al. 2022). The *CCD* gene family has been identified and investigated in many plant species such as *Arabidopsis* (Tan et al. 2003), watermelon (Cheng et al. 2022), pumpkin (Cheng et al. 2022), wax gourd (Cheng et al. 2022), Bottle gourd (Cheng et al. 2022), rapeseed (Zhou et al. 2020) and cotton (Zhang et al. 2021). The function of CCD genes in sunflower has yet to be determined. *Arabidopsis thaliana* has been extensively studied for *CCO* genes, but it lacks Carotenoid Cleavage Dioxygenase Like (CCDL) proteins, limiting its utility for studying CCD enzymes and their functions. *Citrullus lanatus* and *Cucumis melo* are found to have *CCDL* genes that encode the proteins belonging to the CCD enzyme family (Cheng et al. 2022). This family plays an important role in the production and degradation of carotenoids, pigments that provide vibrant colors in fruits and vegetables (Saini et al. 2015). *CCDL* genes in *C. lanatus* and *C. melo* make them appropriate for further research on the CCD enzymes and their functions.

Sunflower is one of the world’s most extensively grown and important crops. The first complete genome sequence of sunflowers was published in 2013 (León et al. 2013). The genome was relatively large, approximately 3.5 Gb, and contained around 31,000 protein-coding genes (Stricevic et al. 2011). Information about *CCO* genes present in sunflower and their role in plant development is completely lacking (Sharma and Shadakshari 2021). *NCED* genes encode enzymes that produce abscisic acid and developmental and stress hormones. CCD enzymes catalyze the cleavage of carotenoids, generating apocarotenoids such as volatile compounds, pigments, and signaling molecules (Meng et al. 2021). Expanding research on *NCED* and *CCD* genes could help further understand their specific roles in sunflower development and physiological processes. It would contribute to a better genetic understanding of sunflower development and provide the potential for breeding and genetic engineering approaches to improve sunflower crops. This research also sheds light on the functional differentiation and evolutionary history of *CCO* genes in plants. Our primary objective was to identify the function and expression patterns of the *CCO* genes in the sunflower genome. We also employed RNA-seq data and various bioinformatics tools to explore their functions. The genome-wide identification and characterization perfoermed in this study will serve as a foundation for the cloning and functional analysis of these genes.

## Materials and methods

### Identification of the *CCO* gene family in H. annuus

The CCO protein sequences of *Arabidopsis thaliana* were obtained from the online database Phytozome v.13 (https://phytozome-next.jgi.doe.gov/). Meanwhile, the Hidden Markov Model (HMM) profile (PF03055) of the RPE65 domain and sequence was acquired from the PFAM database (http://pfam.xfam.org/) (Lu et al. 2020). PF03055 sequence was utilized as a query sequence to search for possible CCO protein sequences against *H. annuus* in the genome database at Phytozome v13 (https://phytozome-next.jgi.doe.gov) utilizing the BLAST-P (Protein-basic local alignment search tool). Amino acids sequences were validated using the default parameters on the NCBI CDD (Conserved Domain Database) (http://www.ncbi.nlm.nih.gov/Structure/cdd/wrpsb.cgi) and Motif finder database (Finn et al. 2011).

### Physiochemical properties and subcellular localization determination

Physiochemical characteristics of *HaCCO* genes, such as protein length (amino acid residues), molecular weight, isoelectric point, Instability Index, and GRAVY, were retrieved using the ProtParam program (http://web.expasy.org/protparam/) (Horton et al. 2006).

The phytozomeV3 database was used to collect gene IDs, chromosomal locations, directions, protein sequences, and CDS of the candidate genes (Gasteiger et al. 2005). The online program WoLF PSORT (https://wolfpsort.hgc.jp/) was utilized to predict the subcellular localization of *HaCCO* (Horton et al. 2006). The heat map was constructed in TBTools for visual inspection from output data that contain localization features in different organelles of cells (Kumar et al. 2018).

### Conserved motif, domain prediction, and intron-exon distribution

The Motif Analysis in the *CCO* gene family proteins was performed using the MEME suite tool (https://meme-suite.org/meme/tools/meme) with default parameters, including 20 motifs (Bailey et al. 2015). Domain analysis of the HaCCO proteins *was* performed using the NCBI Conserved Domain Search (https://www.ncbi.nlm.nih.gov/Structure/cdd/wrpsb.cgi ) using default settings (Islam et al. 2023).

To investigate the distribution of exons and introns, a web tool called the gene structure display server (GSDS) (http://gsds.cbi.pku.edu.cn/) was utilized with both genomic and CDS sequences of the *HaCCO* gene family (Chen et al. 2020a), (C. Chen et al. 2020b).

### Phylogenetic analysis of the *CCO* gene family

MEGA-11 software was used to compare the complete amino acid sequences of the *CCO* gene family in several plant species, including *H. annuus* (Sunflower), *S. lycopersicum* (Tomato), *C. lanatus* (Watermelon), *C. melo* (melon), and *A. thaliana* (Wen et al. 2016). The resulting phylogenetic tree was generated based on the full-length amino acid sequence of the *CCO* genes in *H. annuus* (Ha), *A. thaliana* (At), *and S. lycopersicum (Sl)*. The amino acids were aligned using Muscle (Multiple Sequence Alignment). Using the neighbor-joining (NJ) method, 1000 bootstrap tests, and paired deletion, the phylogenetic tree was created to show the evolutionary relationship between the *CCO* gene family (Gardiner 2010; Iwamoto 2016; Konishi 2007; Miyashima 2019), and then the aligned data was exported to iTol ( https://itol.embl.de/personal_page.cgi) for colorful visualization.

### Chromosomal locations, gene duplication and synteny analysis

Utilizing the available sunflower genome information from Phytozome, the TBtools program was employed to visualize the chromosomal positions and duplications of *CCO* genes. Subsequently, the KaKs calculator within TBtools was utilized to compute the synonymous substitution rate (Ks), non-synonymous substitution rate (Ka), and the Ka/Ks ratio among the duplicated gene pairs. A well-established formula T = Ks/2λ (where λ = Ks/2*(1.5*10^-8) was used to measure the evolutionary divergence (Blanc and Wolfe 2004). Adobe Illustrator CC 2021 was used to modify the output graphs. Sunflower-Arabidopsis and Sunflower-Tomato were analyzed using the Multiple Collinearity Scan toolkit (MCScanX), and the Dual Synteny Plott function in TBtools was utilized to create the graphs for the syntenic analysis (Bettaieb and Bouktila 2020). To exhibit the syntenic relationship of the orthologous *CCO* genes of sunflower, Advanced Circos view software in TBtools was used to construct a map to show a relationship (Ramirez-Tejero et al. 2020).

### *Cis‑*regulatory elements analysis and function determination

To extract *cis*-regulatory elements, 1500 upstream promotor regions of *HaCCO* were used. The PlantCARE (http://bioinformatics.psb.ugent.be/webtools/plantcare/html/) web tool retrieves 5 to 20 bp putative *cis*-elements for the promotor region. The measured cis-regulatory elements were visualized in a heatmap with the help of TBtools (Bulow and Hehl 2016; Jones and Vandepoele 2020).

### Enrichment analysis

ShinyGO v0.75: Gene Ontology Enrichment Analysis + more ( http://bioinformatics.sdstate.edu/go/) was utilized to obtain gene ontology (GO) annotation for sunflower by entering potential candidate protein or gene IDs. A p-value cut-off (FDR) of 0.01 was used to calculate the level of GO enrichment (Bu et al. 2021).

### Transcriptome analysis

The transcriptomic data of organ-specific gene expression (Wu et al. 2023) and the expression profiles of sunflower varieties SF01, SF02, SF03, SF04, SF05, SF06, SF07, SF08, SF09, SF10, SF11, SF12 and SF13 were extracted from the NCBI GEO database (Gody et al. 2020) to investigate the drought stress expression profile of the *CCO* gene family (https://www.ncbi.nlm.nih.gov/geo/). Thirteen sunflower genotypes were chosen to represent the genetic diversity within cultivated sunflowers. Statistix 8.1 pairwise comparison tool was used to enhance understanding of up/down-regulated gene expression by identifying significant changes between conditions, aiding in identifying potential targets for future studies (Gody et al. 2020) (Gody et al. 2020).

### Protein-protein interaction

STRING v11.0 ( https://string-db.org/) was used to analyze Protein-Protein Interactions (PPI) with a high confidence score of 0.7 (Ferrando and Solomon 2021). Our functional enrichment study used a 0.01 threshold. The PPI network was created by combining active interactions with an interaction score of > 0.4 from various sources, including text mining, studies, gene fusion, databases, and co-expression. The interactome map facilitated the identification of key candidate genes responsible for both the physical and functional aspects of this interaction (Xie et al. 2020).

### Target site prediction and validation for miRNA

The identification of the miRNA targeting the 21 *CCO* genes in sunflower was achieved by utilizing the PmiREN website (https://www.pmiren.com/). The CDS of the 21 genes was then compared to the mature miRNA using the PsRNA online server tool (https://www.zhaolab.org/psRNATarget/) using the default setting (Riolo et al. 2020) (Shafiq et al. 2024). The Cytoscape program (https://www.omicshare.com/tools/) was created to access a connection between the predicted miRNA (Liu and Wang 2019), (Sami et al. 2023b).

## Results

### Physiochemical properties and subcellular determination

The pI (isoelectric point), MW (molecular weight), GRAVY (grand average of hydropathy), instability index of the 21 *CCO* genes, and other physical and chemical characteristics are described in (Table 1). The length of the *HaCCO* peptide ranged from 152 (*HaCCDL7*) to 613 (*HaCCD11*) amino acid residues, and the associated MW ranged from 17296.23 to 69410.86 Da. The isoelectric point values ranged from 5.41 (*HaCCDL10*) to 9.44 (*HaCCDL7*).

All HaCCO proteins were found to be hydrophilic based on the negative values obtained from the GRAVY results. The GRAVY value of *HaCCDL7* is 0.026 and is still considered hydrophilic, as it is closer to neutral. A value of 0.026 suggests slight hydrophilicity of the protein, indicating a preference for interaction with water and polar solvents over non-polar solvents. It is essential to recognize that the GRAVY score is just one measure of a protein’s hydropathy and should be considered alongside other factors for a comprehensive analysis of protein properties and functions. The GRAVY score ranges from highly hydrophilic (negative scores) to highly hydrophobic (positive scores), with a score of zero indicating a balance between hydrophilic and hydrophobic residues in the protein sequence.

The instability index of HaCCO proteins demonstrated that the proteins exhibited different levels of stability, with most of them showing instability (instability index > 40). However, a significant portion of the proteins (15 out of 21) were identified as stable (instability index < 40). The most unstable protein was *HaNCED5*. Other unstable proteins were *HaCCD2*, *HaCCD11*, *HaNCED15*, *HaNCED16* and *HaCCD20*.

**Table 1:** Physiochemical properties of the *HaCCO* gene family

Gene ID	Accession	Chromosome		Direction	Size (AA)		pI	Mw	Instability Index	GRAVY
Name	Phytozome ID	no.	Location (Base pairs)		mRNA (CDS)	Peptide		(Da)		
*HaNCED1*	HanXRQChr10g0301261	10	161983332.161985151	R	1779	592	5.9	65303.9	38.74	-0.28
*HaCCD2*	HanXRQChr16g0524481	16	153942754.153945230	F	972	323	6.12	37413.92	42.02	-0.26
*HaCCD3*	HanXRQChr16g0531971	16	183023700.183027192	F	1794	597	6.43	66785.29	39.03	-0.236
*HaCCD4*	HanXRQChr16g0524461	16	153858183.153875270	F	1413	470	8.96	53826.43	35.4	-0.235
*HaNCED5*	HanXRQChr04g0114331	4	126335282.126337046	F	1764	587	6.24	65349.41	44.16	-0.287
*HaCCD6*	HanXRQChr09g0254251	9	124461946.124464016	F	1713	570	6.53	63774.96	29.44	-0.204
*HaCCDL7*	HanXRQChr13g0401841	13	93083247.93083976	F	459	152	9.44	17296.23	29.92	0.026
*HaCCD8*	HanXRQChr13g0399061	13	81577092.81579909	R	1791	596	6.42	66681.64	30.67	-0.134
*HaCCDL9*	HanXRQChr13g0401881	13	93290532.93303998	R	1767	588	5.93	66364.69	39.9	-0.252
*HaCCDL10*	HanXRQChr13g0401851	13	93087583.93095196	F	819	272	5.41	31140.49	35.73	-0.231
*HaCCD11*	HanXRQChr13g0422761	13	184631456.184638343	F	1842	613	6.08	69410.86	40.97	-0.316
*HaCCD12*	HanXRQChr17g0562991	17	169800946.169807303	R	1632	543	5.82	61021.10	32.84	-0.271
*HaCCDL13*	HanXRQChr17g0538671	17	17661688.17669699	R	654	217	5.90	25088.54	28.37	-0.392
*HaCCDL14*	HanXRQChr17g0538661	17	17659198.17661638	R	702	233	6.18	26289.15	24.26	-0.263
*HaNCED15*	HanXRQChr15g0480901	15	64124171.64126954	F	1719	572	6.35	64046.22	43.78	-0.282
*HaNCED16*	HanXRQChr15g0492301	15	136149925.136151710	R	1785	594	5.85	65736.71	41.74	-0.248
*HaCCD17*	HanXRQChr02g0050571	2	143584021.143587868	F	1662	553	5.50	61393.76	33.06	-0.279
*HaNCED18*	HanXRQChr02g0059541	2	179037096.179038851	F	1755	584	7.61	64776.21	36.87	-0.28
*HaNCED19*	HanXRQChr11g0329931	11	40785921.40787682	F	1761	586	6.51	64537.79	39.97	-0.246
*HaCCD20*	HanXRQChr08g0227631	8	90476375.90479305	F	1779	592	5.70	64803.8	43.07	-0.155
*HaCCD21*	HanXRQChr00c0025g0571121	0	19619.23105	R	1674	557	6.05	62082.38	35.07	-0.33

**Fig. 1:**
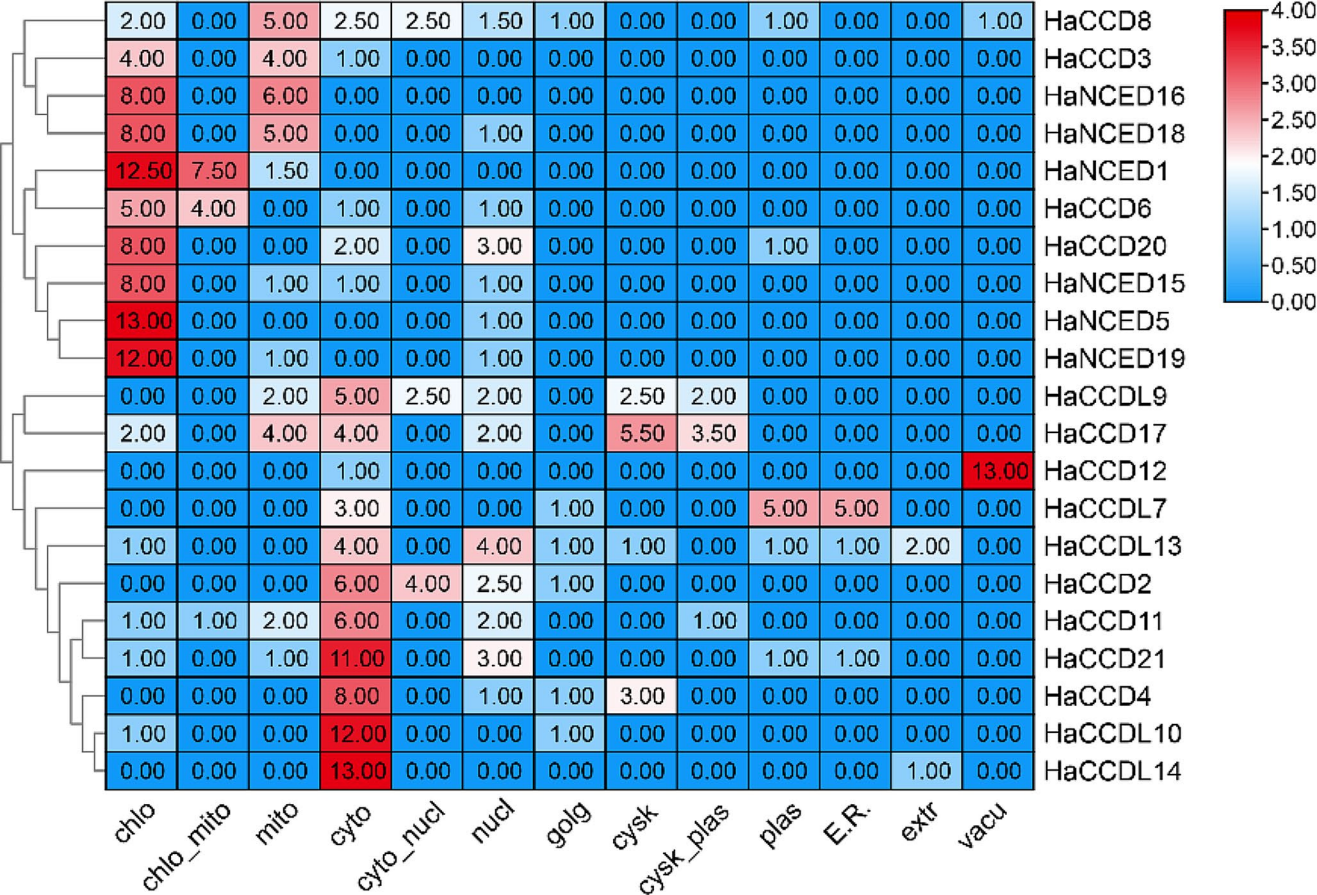
Subcellular localization prediction analysis of HaCCO proteins primarily localized in chloroplasts and cytoplasm. The red colour indicates the maximum number of localizations

A subcellular localization prediction analysis for the CCO proteins was conducted to gain deeper insights into the roles and functions of HaCCO proteins. The maximum number of CCO proteins were localized in chloroplast (86.5) followed by cytoplasm (80.5). The WoLF PSORT tool predicts the subcellular localization of proteins using a variety of factors, including sorting signals, amino acid composition, and functional motifs (such as DNA-binding motifs). It transforms the protein amino acid sequences into numerical localization characteristics (Fig. 1).

### Conserved motif analysis and domain prediction

**Fig. 2:**
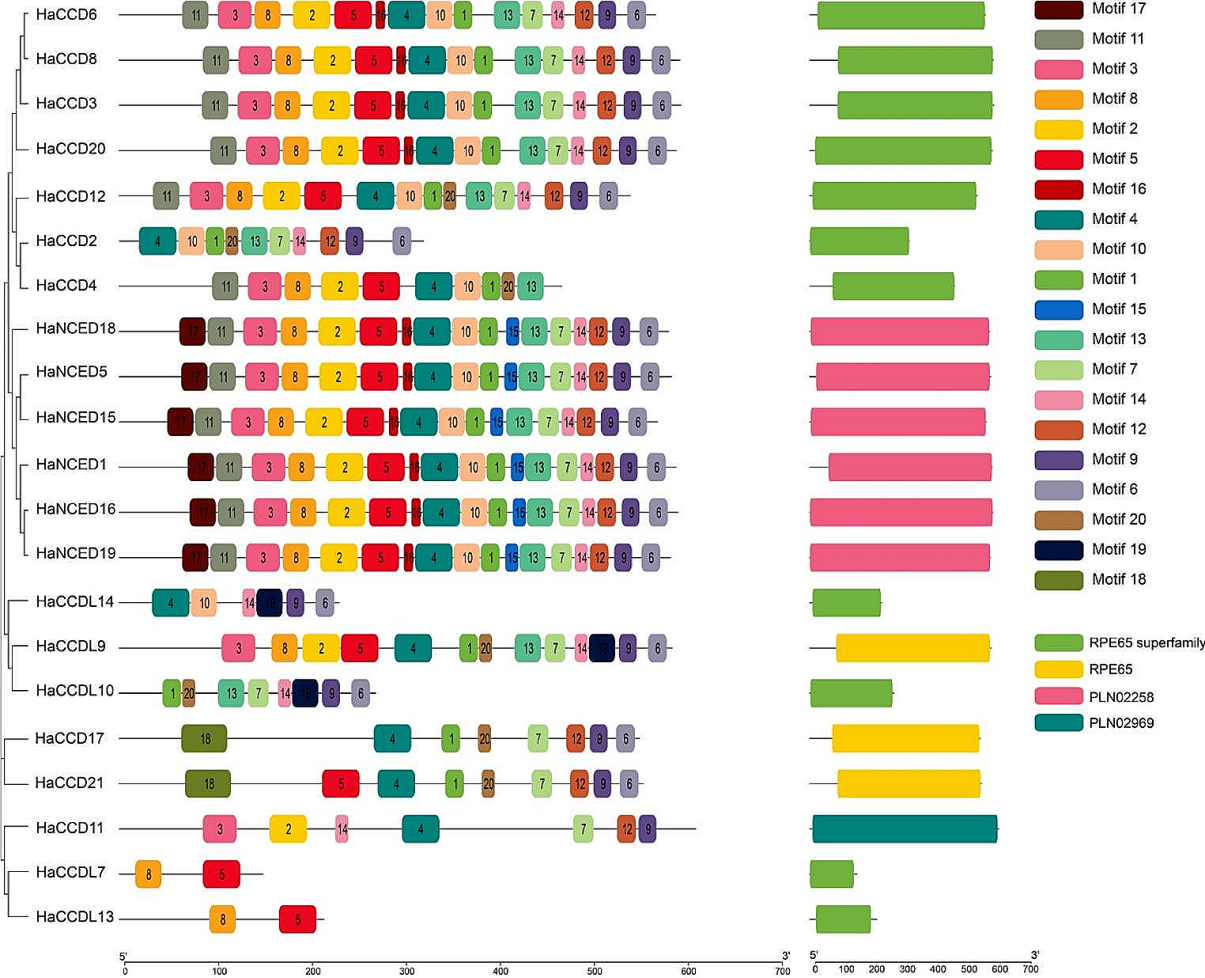
A color-coded bar graph showing the results of a motif distribution analysis of sunflower HaCCO proteins using MEME version 5.5.2 indicates 20 different motifs. The bar graph is connected to a phylogenetic tree to show the relationship between HaCCO proteins and motif distribution

The MEME tool investigated the discovery and distribution of 20 motifs within the *HaCCO* (S Fig. 1). While investigating conserved domains, RPE65, PLN02258, and PLN02969 emerged as subfamilies within the RPE65 superfamily. The *HaCCD2, HaCCD3, HaCCD4, HaCCD6, HaCCD8, HaCCD12, HaCCD17, HaCCD20, HaCCDL7, HaCCDL10, HaCCDL13*, and *HaCCDL14* genes contained the RPE65 superfamily, while *HaCCDL9, HaCCDL17*, and *HaCCD21* constituted the RPE65 subfamily. All NCED proteins were grouped under the PLN02258 classification, including *HaNCED1, HaNCED5, HaNCED15, HaNCED16, HaNCED18*, and *HaNCED19* genes. Additionally, *HaNCED11* was affiliated with the PLN02969 subfamily in a separate classification (Fig. 2). This observation confirms the conservation of the domain across all these proteins. (S Fig. 2). The motifs (13, 6, 14, and 9) showed conservation within the RPE65 superfamily in the analysis of conserved motifs. In particular, a conserved pattern was seen in RPE65 itself (4, 1, 20, 7, 9 and 6). Furthermore, there was a conservation of the pattern (17, 11, 3, 8, 2, 5, 4, 10, 1, 16, 15, 13, 7, 14, 12, 9 and 6) in the PLN02258 subfamily. The analysis of the *CCO* gene family revealed that the genes within the same group shared similar motifs, indicating that these conserved motifs are likely involved in specific activities within a particular group or subgroup. The existence of comparable motifs among various members of the *CCO* gene family implies that gene expansion likely played a role in the evolution of these genes. The *CCDL* family contained a smaller number of motifs than other gene families like *NCED* and *CCD*. The *HaCCDL7*, *HaCCDL13* and *HaCCDL10* had 2, 2 and 8 motifs, respectively (Fig. 2).

### Intron-exon distribution

**Fig. 3:**
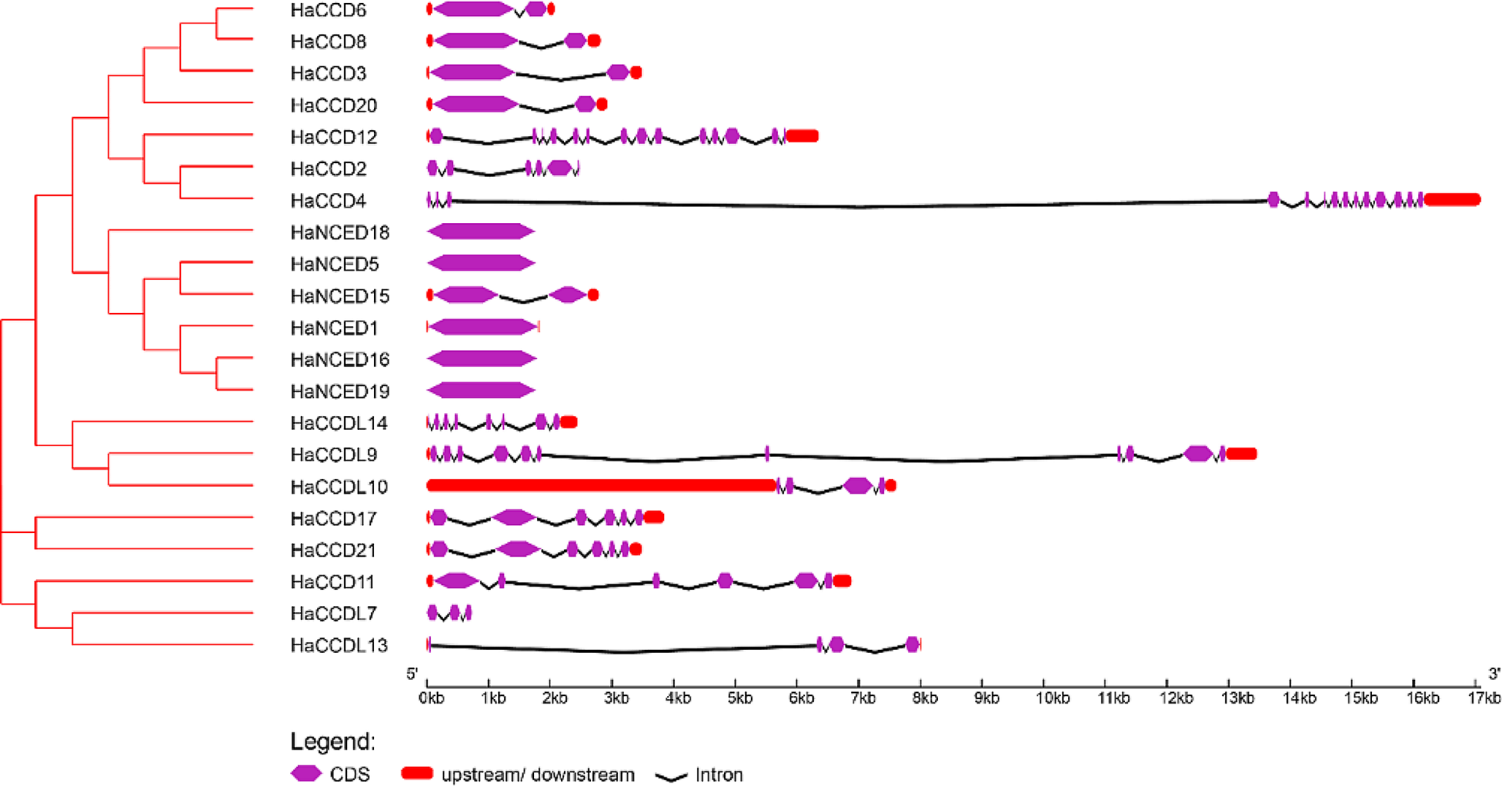
The phylogenetic relationships and gene structures of *HaCCO* genes. The phylogenetic tree was constructed using the full-length sequences of *HaCCO* genes. Different colours indicate the intron-exon; purple shapes represent exons, red shapes represent UTR regions, and black lines represent introns

The fundamental structure of genes and the evolutionary relationships between genes or species are influenced by the placement of exons and introns. Exon and intron count and distribution patterns explain a gene family’s evolutionary background. There was a clear correlation between the two when the exon-intron structure of the *HaCCO* genes and their phylogenetic relationships were analyzed. *HaCCO* genes ranged in intron count from one in *HaNCED18* to fourteen in *HaCCD4*. All gene sequences contained exons and introns (Fig. 3).

### Phylogenetic analysis and classification of the *CCO* gene family

**Fig. 4:**
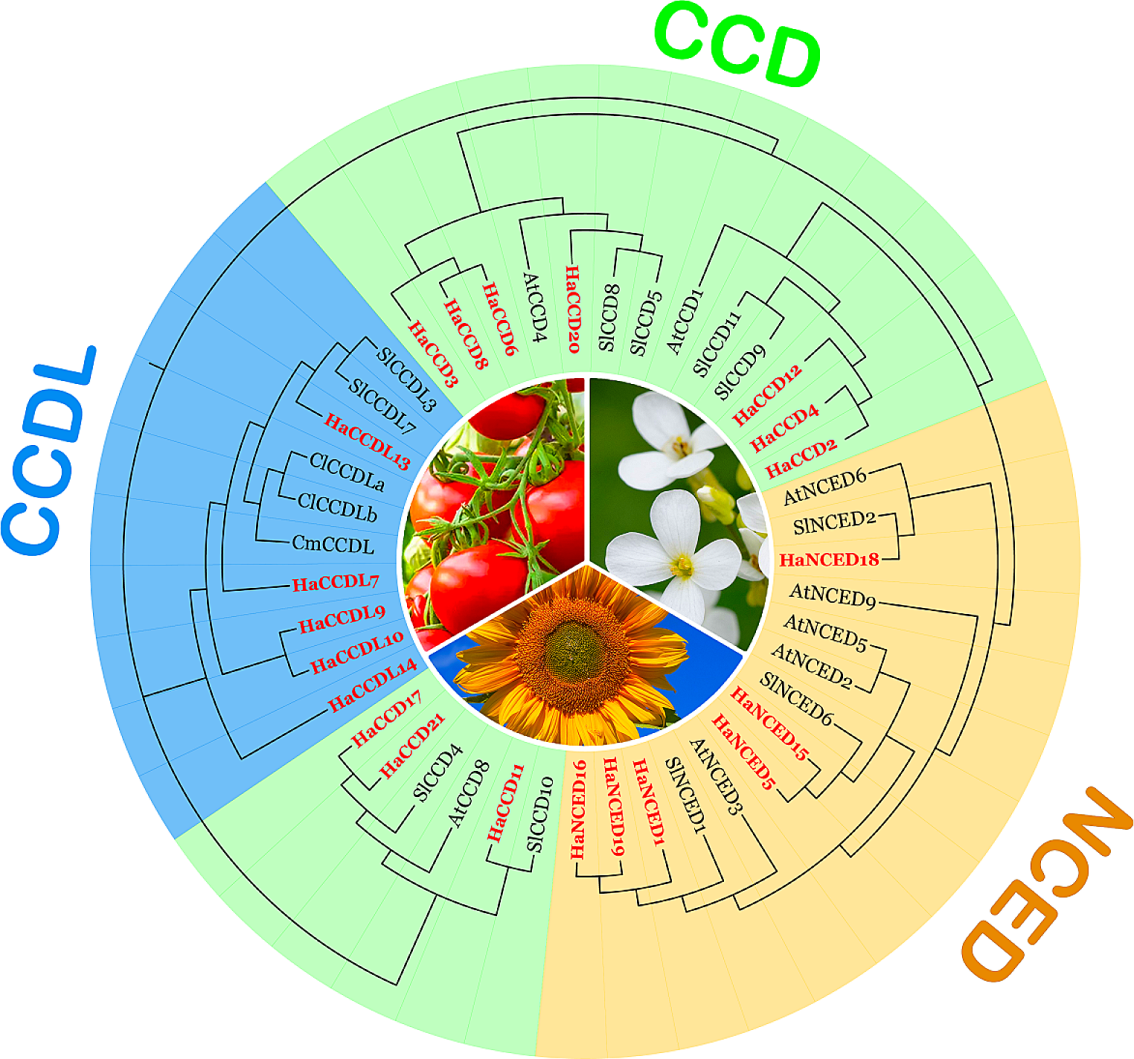
Phylogenetic relationship among the *CCO* gene family of *Helianthus annuus*, *Citrullus lanatus*, *Cucumis melo*, *Arabidopsis thaliana*, and *Solanum lycopersicum* were studied. Red stars are used to identify the *H. annuus* gene. In MEGA 11, evolutionary analyses were carried out and further edited with Adobe Illustrator 2022 CC

To investigate the evolutionary relationships, 43 CCO proteins from 5 species *H. annuus* (21), *S. lycopersicum* (11), *C. lanatus* (2), *C. melo* (1) *and A. thaliana* (8) were used to produce a phylogenetic tree. *C. lanatus* and *C. melo* both contain *CCDL* genes, which make them suitable candidates for further study of *CCD* genes. The model plant *A. thaliana* does not contain *CCDL* genes (Cheng et al. 2022). Further, classification results of the *CCO* genes family fell into 3 subfamilies *(CCDL, CCD*, and *NCED*). The following color coding differentiates the subfamilies: Subfamily I is represented by blue, Subfamily II by green and Subfamily III by brown (Fig. 4).

### Chromosomal locations, gene duplication and synteny analysis

**Fig. 5:**
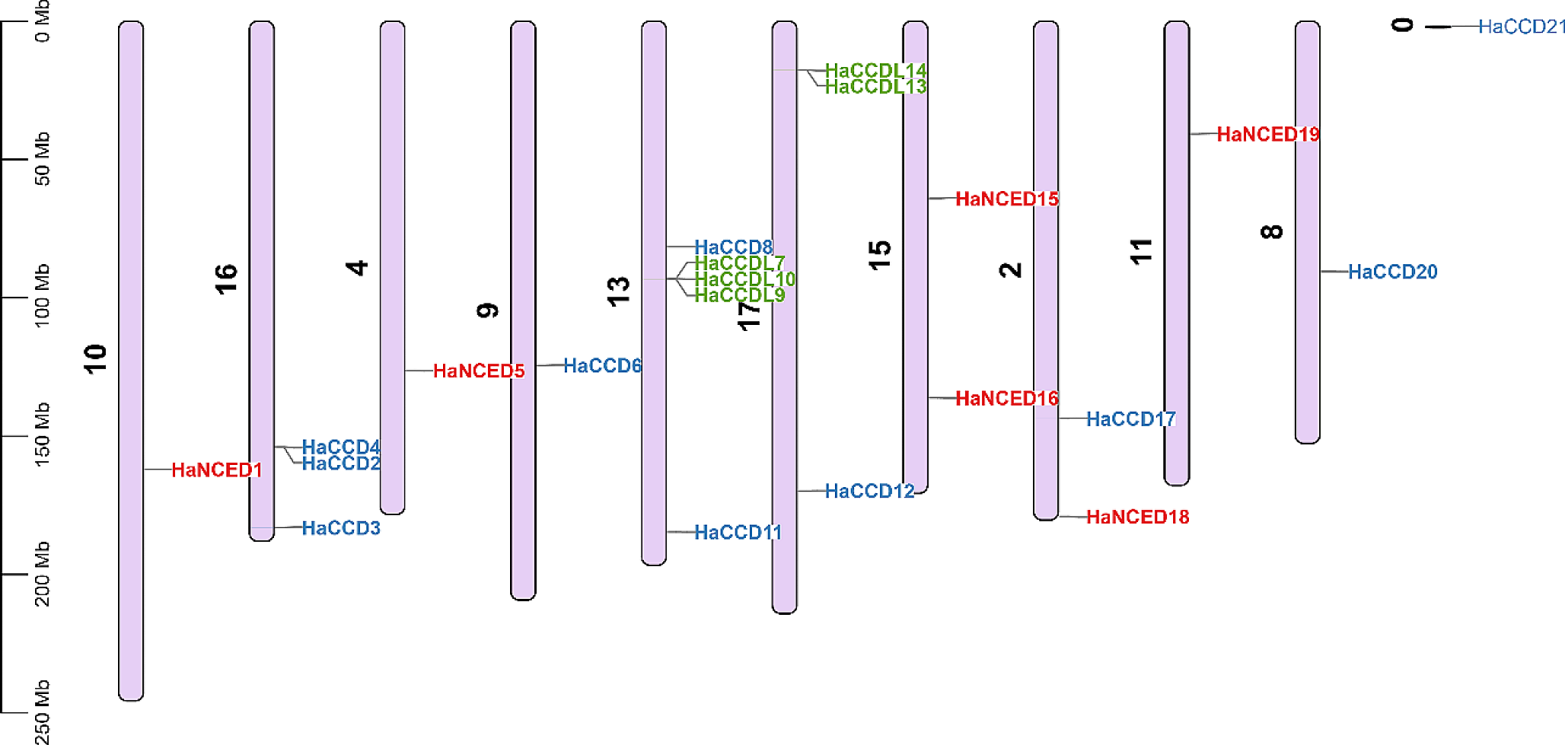
Chromosomal distribution of the *HaCCO* gene family in sunflower. This analysis provides insights into the spatial arrangement and potential interactions among *HaCCO* genes

The physical location of the *HaCCO* genes was identified on different chromosomes, but only *HaCCD21* was located on the scaffold region of the sunflower genome region. Only chromosome numbers 2, 4, 8, 9, 10, 11, 13, 15, 16 and 17 contain *HaCCO* genes. Chromosome 13 had the highest number of genes, i.e., *HaCCDL7*, *HaCCD8*, *HaCCDL9* and *HaCCDL10*. The *CCDL* genes are only present on chromosomes 13 and 17 (Fig. 5).

**Fig. 6:**
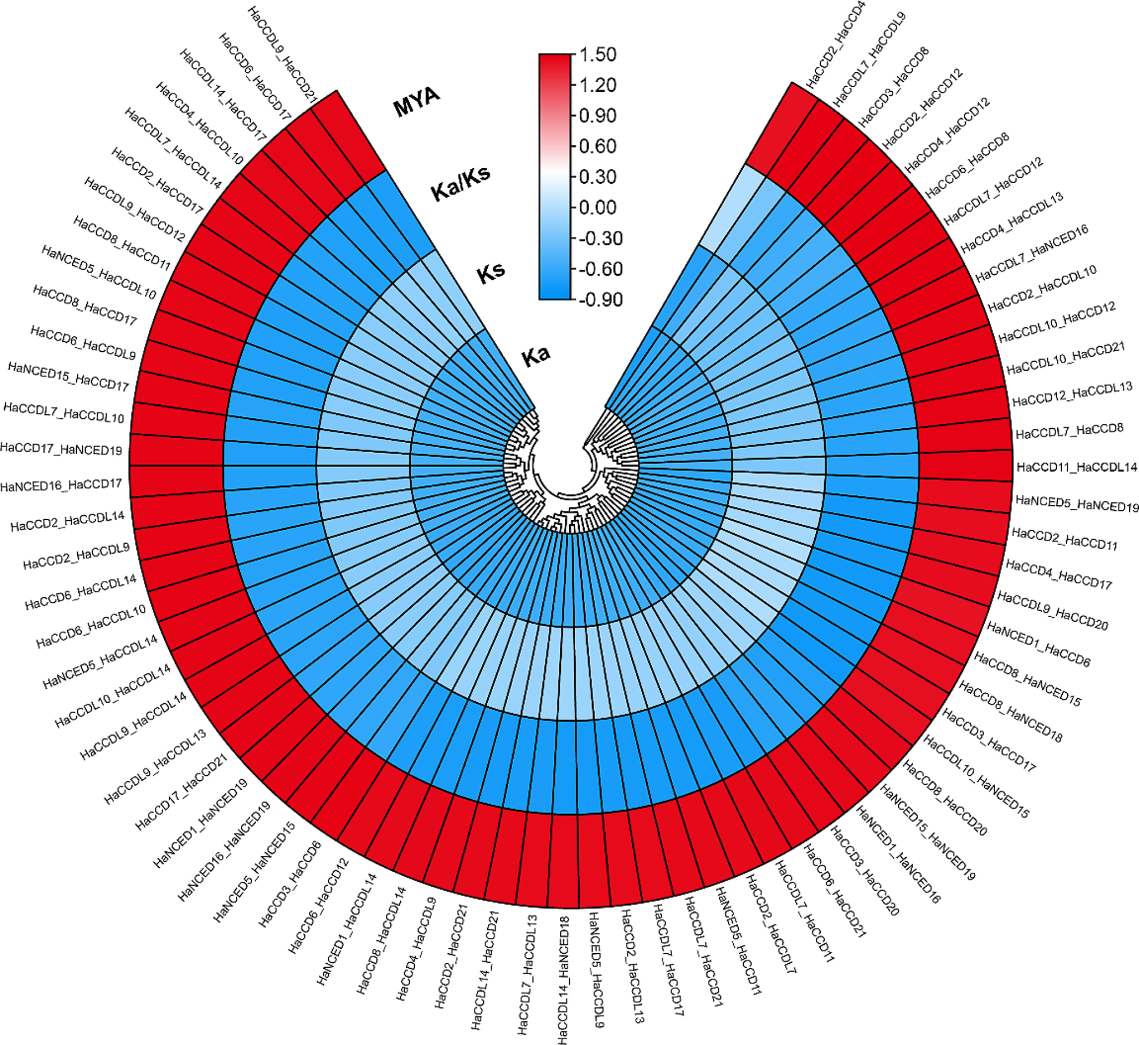
Ks and Ka were estimated using the TBTools and further edited with Adobe Illustrator 2022 CC. The clock-like rate (λ) for sunflower was 1.5 × 10^-8. The date of the duplication event was determined using T = Ks/2λ

The dates of the duplication blocks were then traced using Ks, which was used to estimate the dates of duplication events. Figure 6 compares segmental and tandem duplication blocks. Sunflower’s segmental duplications of the *CCO* genes originated from *HaCCD2_HaCCD4*; 1.51 Mya (Ks = 0.045) to *HaNCED1_HaCCD6*; 197.67 Mya (Ks = 5.93), with the mean being 82.88 Mya (Ks = 2.49); the Ka/Ks of tandem duplications ratio was less than 1 indicates purifying selection, which means that natural selection is acting to preserve the amino acid sequence because it is important for the protein’s function. This type of selection is also known as negative selection, and it operates to eliminate deleterious mutations that may affect protein function (Liu et al. 2019).

Under purifying selection, a protein is anticipated to exhibit a Ka/Ks ratio of less than one due to a higher rate of synonymous substitutions than non-synonymous substitutions. Based on molecular clock estimates using synonymous substitution rates, the estimated divergence time between sunflower and Arabidopsis is around 34 million years ago (Mya) (Xu et al. 2013), while the estimated divergence time between sunflower and tomato is about 90 Mya (Mascagni et al. 2017). According to these calculations, tomato and Arabidopsis are more closely related than sunflower. These findings imply that the segmental/tandem expansion of the sunflower *CCO* family may be traced to recent duplication events and that the duplicated *CCO* genes continued to function.

**Fig. 7:**
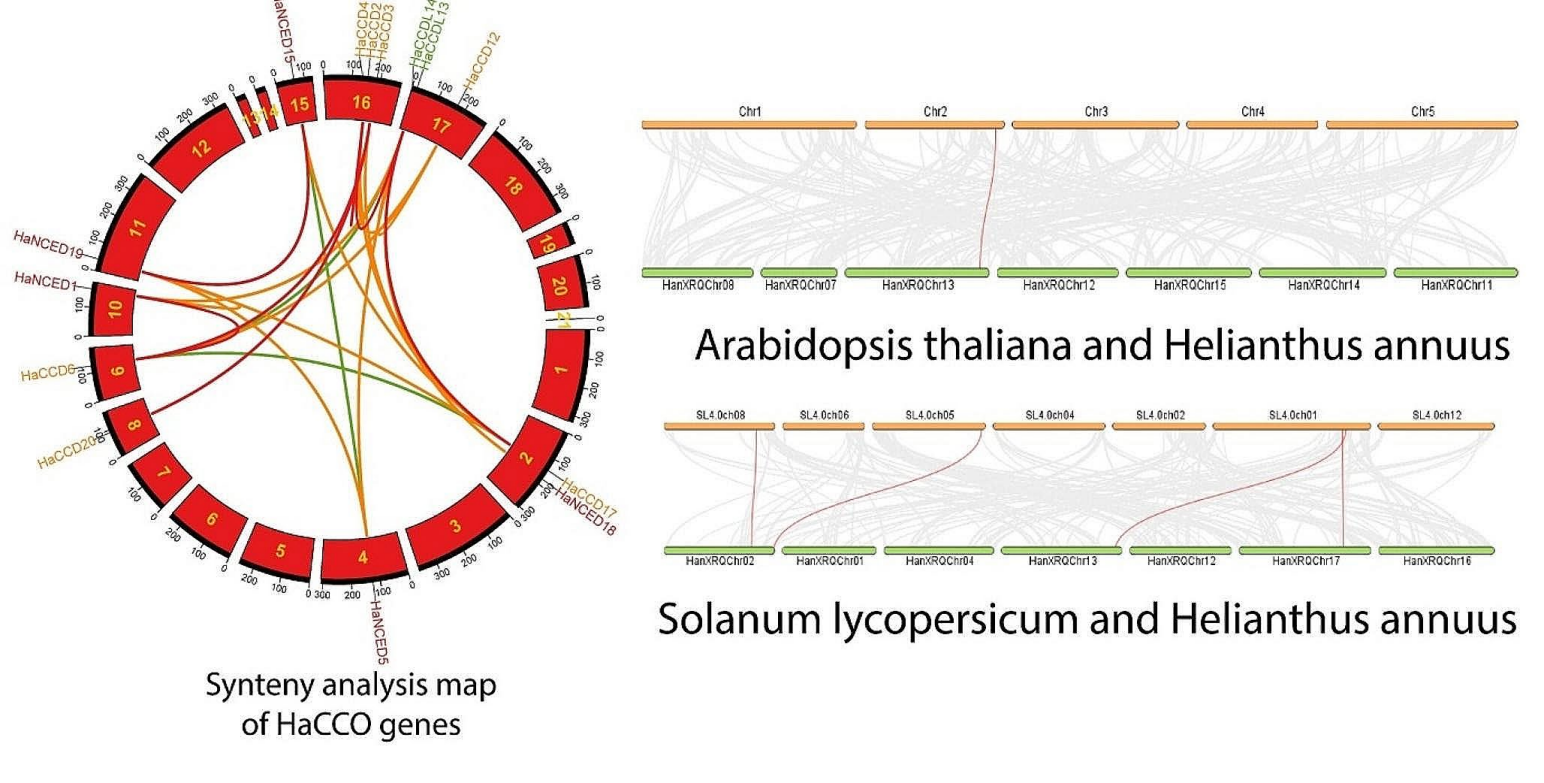
**(a)** Distribution of *HaCCO* genes on sunflower chromosomes, with lines indicating potential gene duplications on different chromosomes. Figure 7**(b)** Chromosomal distribution and intrachromosomal linkages of *CCO* genes between sunflower-Arabidopsis and sunflower-Tomato. The red lines represent duplicate gene pairs, while the gray lines represent all of the synteny blocks in the sunflower genome. The chromosome number at the top of each chromosome indicates that segmental duplication of genes is more common than tandem duplication in the *HaCCO* gene family

Seventy-three pairs of paralogous genes in *HaCCO* were found in our study. These duplicated genes can shed light on how a gene family has expanded, primarily due to the result of tandem and segmental duplications in plants (Fig. 7a). Contiguous homologous genes on a single chromosome with no more than one intervening gene suggest tandem duplication. For instance, *HaCCD17* and *HaNCED18* were near chromosome 2, and *HaCCDL13, HaCCDL14*, and *HaCCD12* were located on chromosome 17. Similarly, *HaCCD4, HaCCD2* and *HaCCD3* were located on chromosome 16. These genes also have comparable gene structures and conserved motifs, implying that they are tandem duplicates.

A chromosome in *A. thaliana* (Chr2) was found to be orthologous to a chromosome in *H. annuus* (HanXRQChr13). This indicates that these two chromosomes are homologous, meaning they shared a common ancestral chromosome and have preserved a comparable gene order and organization throughout evolutionary time. On the other side, three chromosomes in *S. lycopersicum* (SL4.0ch08, SL4.0ch05 and SL4.0ch01) were found to be orthologous to different chromosomes in *H. annuus* i.e. SL4.0ch08 with HanXQChr02, SL4.0ch05 with HanXQChr02, SL4.0ch01 with HanXQChr13 and SL4.0ch01 with HanXQChr17. This meant that these chromosomes are homologous and have similar gene order and organization despite originating from different species (Fig. 7a).

### *Cis*‑regulatory elements analysis and function determination

**Fig. 8:**
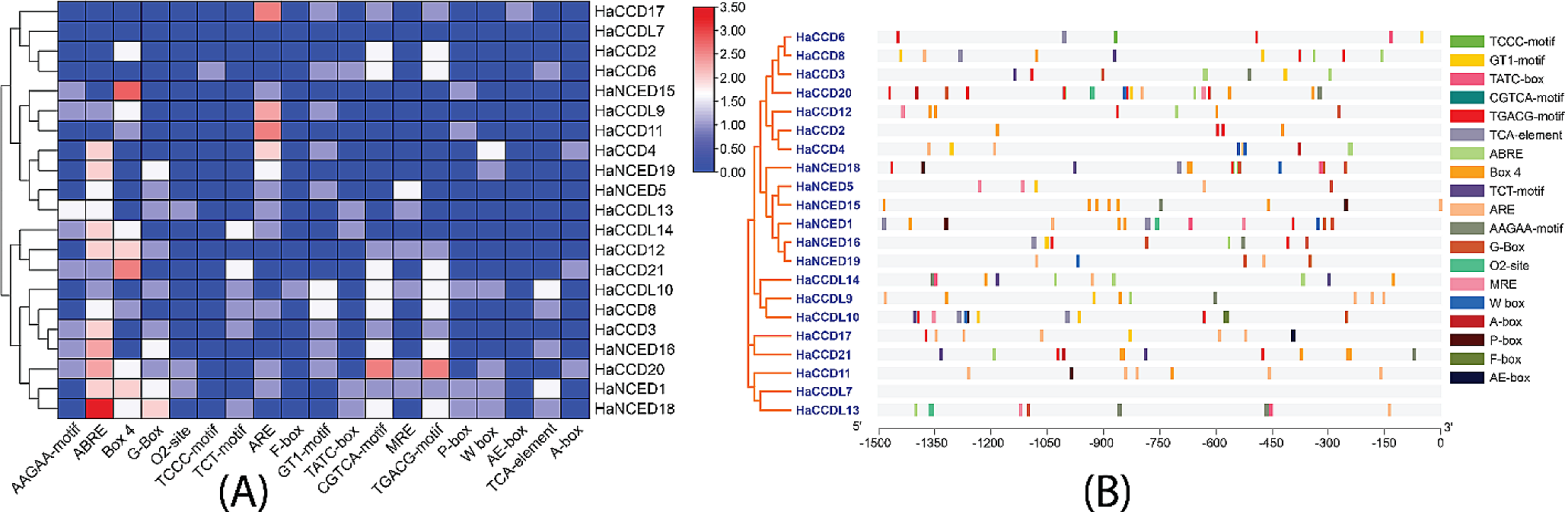
Cis-regulatory element analysis of *HaCCO* genes in sunflower promoter’s process is associated with different plant developmental processes. **(A)** The statistics of *cis*-regulatory elements of each *HaCCO* gene. **(B)** The distribution of cis-regulatory elements on the *HaCCO* gene promoter

A study on the promoter regions of *HaCCO* genes found a total of 230 cis-elements after excluding common elements, such as the TATA-box and CAAT-box, as well as unidentified functional elements (Fig. 8).

Among *HaCCO* genes’ elements, the analysis identified the predominant group as the light-responsive category, constituting 213 elements (51.82%). This group encompassed motifs such as Box 4, MRE, and G-box elements. The second largest group was the plant hormones-related group, comprising 103 (25.06%) elements and including motifs such as CGTCA-motif and TGACG-motif for MeJA response, TCA-element for SA response, GARE-motif, TATC-box, and P-box for GA response, ABRE for ABA response, and TGA-element for auxin response. Additionally, the analysis revealed a stress response group comprising 60 (14.62%) elements and a development-related response group comprising 11 (2.68%) elements.

Within the light-responsive group, the highest counts were observed for Box 4 with 29 elements and G-box with 15 elements. Among the plant hormone-related groups, the highest count was observed for ABRE with 44 elements. These findings provide valuable insights into the distribution of response elements in the analyzed dataset, which may have implications for understanding the regulatory mechanisms involved in plant growth, development, and stress responses (Table 2).

**Table 2:** Functions, Length, Direction and Sequences of *HaCCO cis*-elements

Cis-Element	Sequence	Length	Direction	Function
AAGAA-motif	GGTAAAGAAA	10	+	interactions with specific transcription factors or RNA-binding proteins
ABRE	ACGTG	5	-	cis-acting element involved in the abscisic acid responsiveness
Box 4	ATTAAT	6	+	part of a conserved DNA module involved in light responsiveness
G-box	CACGTC	6	+	cis-acting regulatory element involved in light responsiveness
O2-site	GATGA(C/T)(A/G)TG(A/G)	10	+	involved in zein metabolism regulation
TCCC-motif	TCTCCCT	7	-	part of a light responsive element
TCT-motif	TCTTAC	6	-	part of a light responsive element
ARE	AAACCA	6	+	essential for the anaerobic induction
F-box	CTATTCTCATT	10	+	involved in cellular development
GT1-motif	GTGTGTGAA	9	-	light responsive element
CGTCA-motif	CGTCA	5	-	involved in the MeJA-responsiveness
MRE	AACCTAA	7	-	MYB binding site involved in light responsiveness
TGACG-motif	TGACG	5	+	involved in the MeJA-responsiveness
P-box	CCTTTTG	7	-	gibberellin-responsive element
W box	TTGACC	6	+	inducing plant genes in response to pathogen attack and acts as a binding site for WRKY TFs
AE-box	AGAAACTT	8	+	part of a module for light response
TCA-element	CCATCTTTTT	10	-	cis-acting element involved in salicylic acid responsiveness
A-box	CCGTCC	6	+	part of a light responsive element

### Go annotation and orthologue identification

**Fig. 9:**
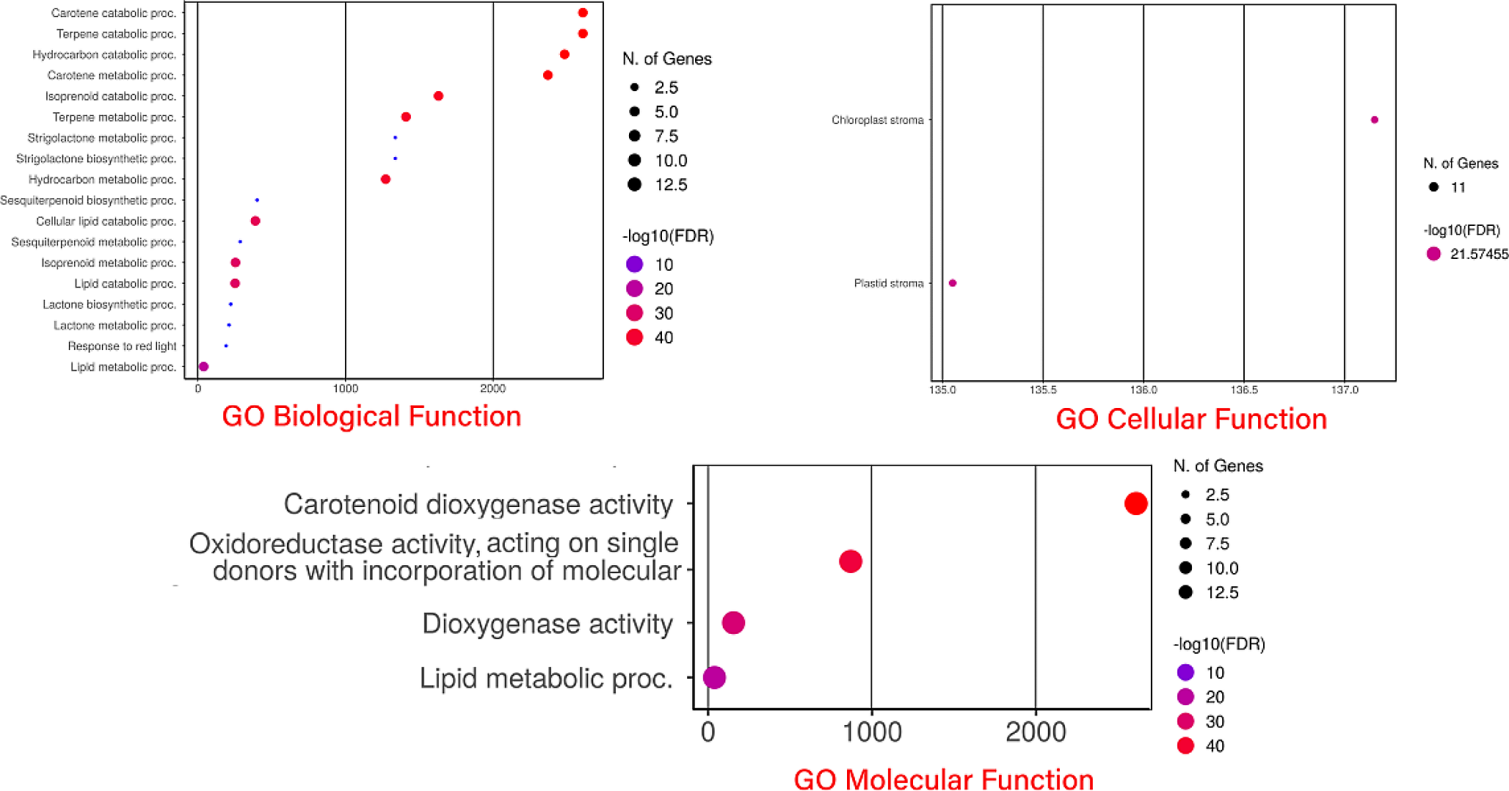
Fold Enrichment chart representing the overlapping *HaCCO* genes functions. Red color dot plots represent the greater number of genes involved in that process and vice versa for small blue sizes

A GO enrichment analysis was conducted to gain a deeper understanding of the functions associated with *HaCCO* genes. The study categorized all CCO proteins’ biological processes, molecular functions, and cellular components (Fig. 9; Table 2). It identified the *A. thaliana* orthologues with *H. annuus* and their corresponding gene expression patterns in the *HaCCO* gene family. In the biological process category, the maximum number of genes of CCO proteins were involved in the carotene metabolic process (GO:0016119) and terpene catabolic process (GO:0046247), respectively. As for their molecular functions, carotenoid dioxygenase activity (GO:0010436) (Wang et al. 2009). Regarding the cellular component, the chloroplast stroma (GO:0009570) was highly enriched, indicating that these HaCCO proteins may play diverse roles in cellular metabolism (S Fig. 3), (See Table 3).

**Table 3:** *Arabidopsis* orthologue of *H. annuus* and their gene expression pattern in the *HaCCO* gene family

Sunflower Gene ID	Arabidopsis Orthologue	Biological Process	Cellular Function	Molecular Function	Location	Gene Expression (Transcriptomic data)	Reference
*HaCCD2, HaCCD4, HaCCDL9, HaCCDL10, HaCCD12*	*AtCCD1*	carotene catabolic process, carotenoid catabolic process	is active in the chloroplast stroma	9-cis-epoxycarotenoid dioxygenase activity, carotenoid dioxygenase activity	Chloroplast	*HaCCD2* (stem and leaves), *HaCCD4* (axils), HaCCDL9 (stem), *HaCCDL10* (roots), *HaCCD12* (highly expressed in leaves)	(Priya et al. 2017)
*HaCCDL14*	*AtNCED2*	Process of synthesizing abscisic acid that contributes to the catabolism of carotene	is active in the chloroplast stroma	enables 9-cis-epoxycarotenoid dioxygenase activity, carotenoid dioxygenase activity	Cytosol	*HaCCDL14* (leaves)	(Chen et al. 2020b)
*HaNCED15, HaNCED16, HaNCED19*	*AtNCED3*	abscisic acid biosynthetic process, hyperosmotic salinity response	is active in the chloroplast stroma	enables 9-cis-epoxycarotenoid dioxygenase activity, carotenoid dioxygenase activity	Chloroplast, Cytosol	*HaNCED15* (stem), *HaNCED16* (leaves), *HaNCED19* (axils and leaves)	(Bader et al. 2023)
*HaCCD3, HaCCD6, HaCCDL7, HaCCD8, HaCCD20*	*AtCCD4*	beta-carotene catabolic process, carotene catabolic process	is active in the chloroplast stroma	carotenoid dioxygenase activity, protein binding	Cytosol	*HaCCD3* (not expressed), *HaCCD6* (flower), *HaCCDL7* (not expressed), *HaCCD8* (leaves), *HaCCD20* (highly expressed in leaves)	(Kim et al. 2022)
*HaCCDL13*	*AtNCED6*	abscisic acid biosynthetic process, response to red light, response to red or far red light	is active in the chloroplast stroma	enables 9-cis-epoxycarotenoid dioxygenase activity, carotenoid dioxygenase activity	Chloroplast	*HaCCDL13* (axils)	(Wang et al. 2023b)
*HaCCD17, HaCCD21, HaCCD11*	*AtCCD8*	leaf morphogenesis, response to auxin, secondary shoot formation,	is active in the chloroplast	enables 9-cis-10’-apo-beta-carotenal cleavage oxygenase activity	Chloroplast	*HaCCD17* (highly expressed in root), *HaCCD21* (root), *HaCCD11* (root)	(Korwin Krukowski et al. 2022)
*HaNCED1, HaNCED5, HaNCED18*	*AtNCED9*	abscisic acid biosynthetic process, involved in the carotene catabolic process	is active in the chloroplast stroma	enables 9-cis-epoxycarotenoid dioxygenase activity, carotenoid dioxygenase activity	Plasma membrane, E.R	*HaNCED1* (axils), *HaNCED5* (axils), *HaNCED18* (not expressed)	(Wang et al. 2023a)

### Transcriptome analysis

#### Drought stress-induced gene expression analysis in sunflower

Thirteen different sunflower genotypes, including inbred lines and their hybrids, were selected to represent the genetic diversity within cultivated sunflower.

**Fig. 10:**
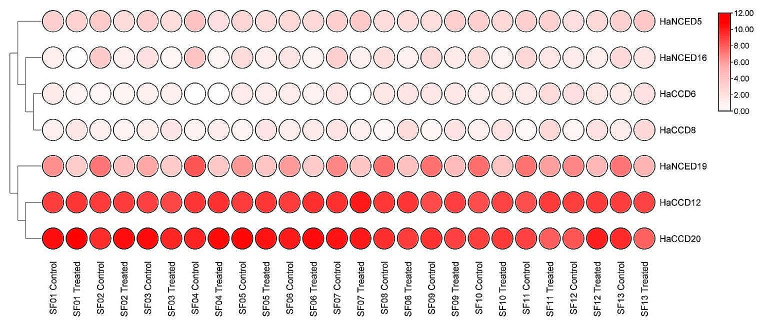
Heat map of the transcriptomic expression profile of sunflower’s *CCO* genes under drought and water conditions. The heat map was further edited with AdobeIllustrator 2022 CC

Based on the RNA-seq data analysis of sunflower data, it was found that the *HaNCED16* and *HaNCED19* were highly expressed and up-regulated. They were involved in the biosynthesis of the hormone ABA (Fig. 10). Due to the differential expression of these genes, the sunflower samples may be actively controlling the production of ABA in response to stress (Table 2).

#### Investigating organ-specific gene expression in sunflower

**Fig. 11:**
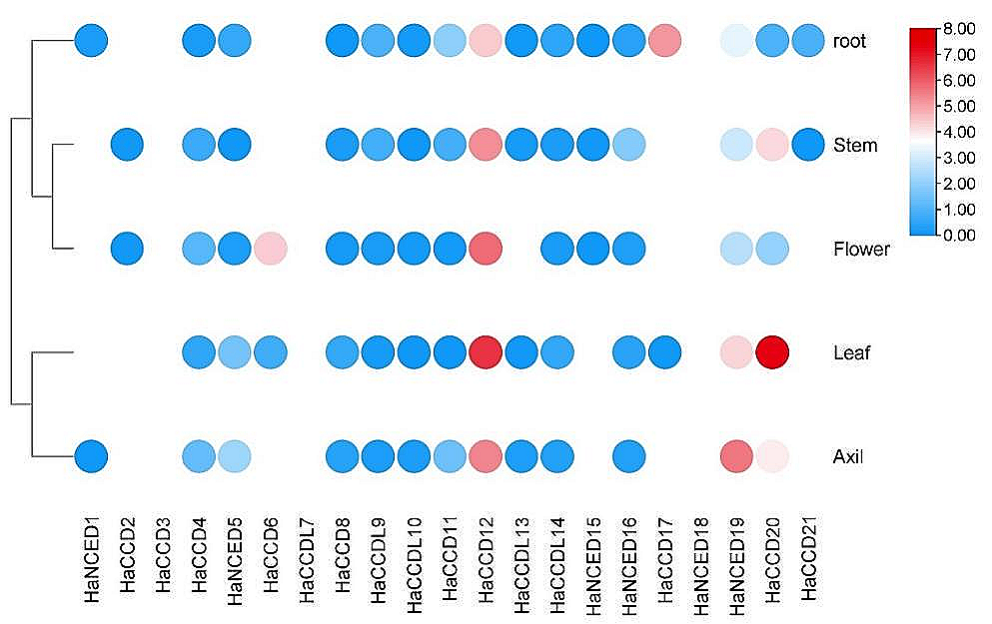
Heat map of sunflower’s transcriptomic expression profile of the *CCO* genes under organ-specific gene expression. The heat map was further edited with Adobe Illustrator 2022 CC

The genes involved in the biosynthesis of abscisic acid and carotenoids exhibited distinct patterns of activity in different plant parts. *HaCCD4, HaNCED5, HaCCD8, HaCCDL9, HaCCDL10, HaCCD11, HaCCD12, HaCCDL14, HaNCED16, HaNCED19* and *HaCCD20* exhibited significance expression levels in all plant parts. *HaNCED1* was primarily active in the root and axil, while *HaCCD2* was slightly expressed in the stem and flower. *HaCCD3*, *HaCCDL7* and *HaNCED18* were not detected in any tissue. *HaCCD6* was only expressed in the leaf and flower, while *HaCCD8* displayed moderate expression throughout most plant parts. *HaCCD17* showed substantial expression in the root and leaf (Fig. 11; Table 2).

### Protein-protein interaction

**Fig. 12:**
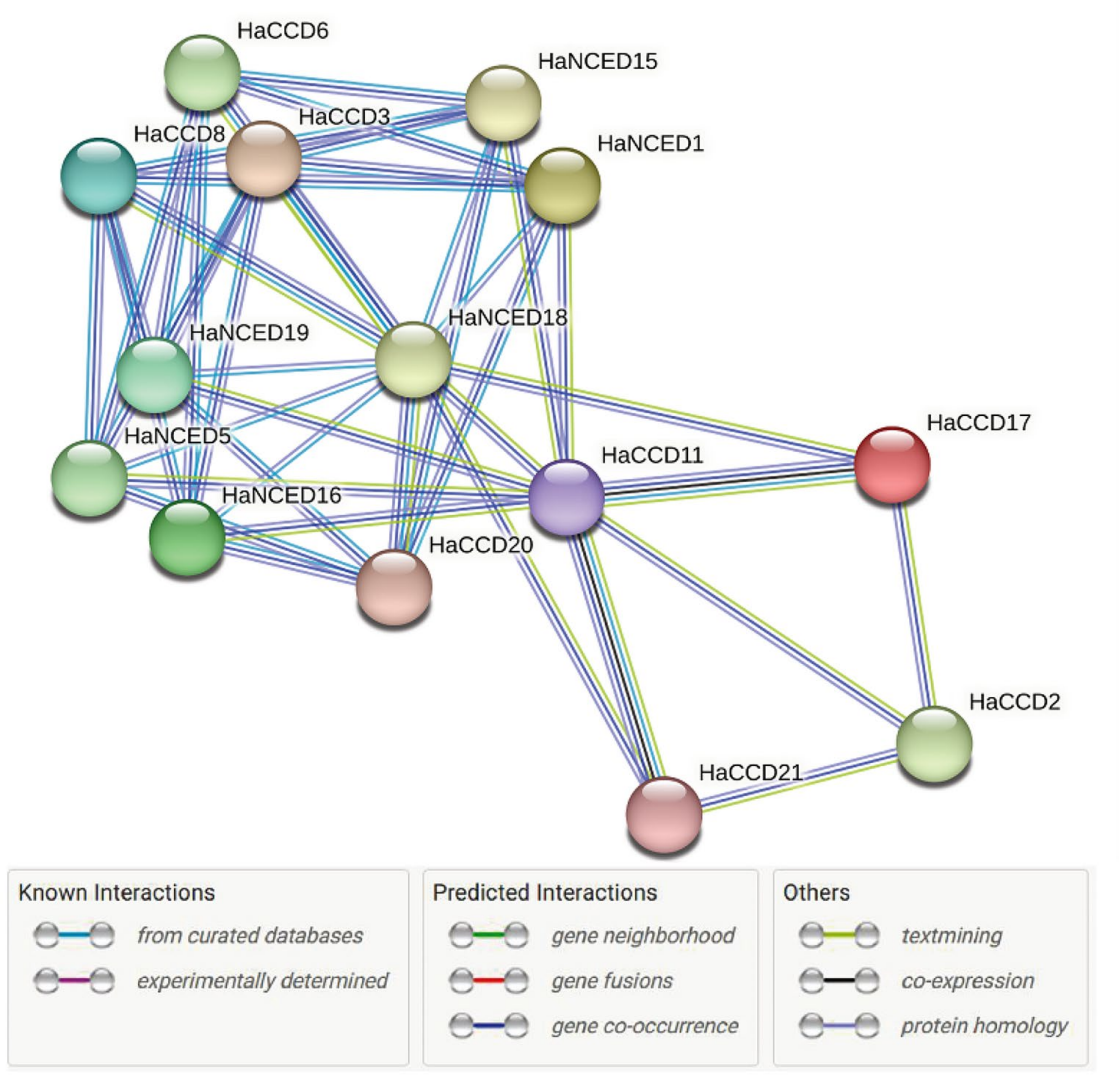
Protein-protein interaction between the genes of *H.annuus*. Each color represents a specific highly significant GO enrichment and colored lines represent various types of interactions. The biosynthesis proteins are predicted via the STRING database

The network comprises 21 nodes (proteins) and 42 edges (interactions), indicating an average of 4 interactions per protein. The average local clustering coefficient is 0.269, suggesting a moderate level of clustering in the network. This indicates that proteins within the network tend to interact with other proteins that are also interconnected. Only 14 out of 21 HaCCO proteins (HaNCED1, HaCCD2, HaCCD3, HaNCED5, HaCCD6, HaCCD8, HaCCD11, HaNCED15, HaNCED16, HaCCD17, HaNCED18, HaNCED19, HaCCD20 and HaCCD21) were interconnected and had an interaction between them. The other 7 proteins (HaCCD4, HaCCDL7, HaCCDL9, HaCCDL10, HaCCD12, HaCCDL13 and HaCCDL14) did not have any interaction between them, (See Fig. 12).

### MiRNA target site prediction and validation

Putative miRNA targets are studied in genome-wide analysis because miRNAs play a crucial role in regulating gene expression. Predicting and validating miRNA targets can provide insights into the molecular mechanisms underlying diseases and can be used to design therapeutic strategies. In our study, 60 miRNAs were found targeting all genes except *HaCCDL7*, *HaCCD8*, *HaCCDL9*, *HaCCDL10* and *HaCCD17*. These miRNAs ranged from 20 to 22 amino acids in length.

7 miRNAs targeted *HaCCD20*, while *HaCCD21* and *HaNCED19* were targeted by 3 miRNAs each. *HaCCD4*, *HaCCDL14*, and *HaCCD3* were targeted by 2 miRNAs each, while 8 miRNAs target *HaCCD6*. *HaCCDL13*, *HaNCED1*, and *HaNCED18* were targeted by 5 miRNAs each, while 13 miRNAs targeted *HaNCED16*. *HaCCD11*, *HaCCD12*, *HaCCD2*, *HaNCED15*, and *HaNCED5* were only targeted by one miRNA each. These miRNAs can regulate the expression of the targeted genes, leading to changes in the biosynthesis and levels of various plant hormones, which can, in turn, affect plant growth, development, and responses to environmental stressors. Consequently, controlling the expression of miRNA-mediated genes was a crucial method for preserving plant adaptability and homeostasis. The miRNAs were specific and targeted only one gene, but multiple miRNAs targeted a single gene. Most miRNAs inhibit cleavage, while others inhibit the translation of their respective targeted genes. Han-miR162a and Han-miR162b inhibit the translation of *HaCCD4*, while Han-miR170d, Han-miR170i, and Han-miR170j inhibit the translation of *HaCCD21*. Han-miRN5681 inhibits the translation of *HaCCD20*, and Han-miRN5742 inhibits the translation of *HaNCED15*, indicating that the miRNA binds to the target mRNA and prevents it from being translated into a protein. All miRNA’s inhibition modes were cleavage, which suggests that this miRNA may downregulate the expression of all mRNAs by causing the degradation of its mRNA (S Table 1).

## Discussion

The *CCO* gene family has been extensively studied in various species in the past few years through bioinformatics analysis. However, research on the *HaCCO* genes family in sunflower has been relatively limited compared to other species, leaving a gap in our knowledge about *CCOs* in sunflower (Priya et al. 2019) (Sami et al. 2024). In this study, 21 *HaCCO* genes in the sunflower genome were identified and characterized and showed different genes and pathways that can be used to prepare resilent sunflower varieties against abiotic stresses.

The *HaCCO* genes in the sunflower genome were studied based on their physicochemical parameters to see how they differed among proteins in the same clade (Cheng et al. 2022). All identified HaCCO proteins were hydrophilic based on negative GRAVY values, indicating a preference for interaction with water with net electrical charges at different pH levels (Priya et al. 2019). The instability index pointed out the instability of six proteins. Analysis of subcellular localization indicated that HaCCO proteins were present in diverse organelles, such as chloroplasts, mitochondria, cytoplasm, cytosol, endoplasmic reticulum, nucleus, and plasma membrane (Ji et al. 2023). Interestingly, more than half of the proteins were localized in the chloroplast (86.5) and cytoplasm (80.5) (52.2%, 167 of 302.5), suggesting that HaCCO proteins may play crucial roles in the function of these organelles.

Comparing genomes between different species can provide valuable information about the evolution and organization of genes (Yao et al. 2022). It also helps to transfer genomic data from a well-studied taxon to a less-studied one (Xu et al. 2013). In this case, we identified 73 pairs of paralogous genes in the genome, which could have arisen through gene duplication events. This duplication can provide useful information about expanding gene families, a common occurrence in plants due to tandem and segmental duplications (Wei et al. 2022b).

Members with comparable subgroups tend to have similar behaviors (Zhou et al. 2019). As a result, phylogenetic analysis might aid in advancing functional genomics. In this study, 43 CCO proteins were identified, possessing complete domain sequences (Wei et al. 2022a). These proteins were categorized into three subfamilies based on their sequence structures and phylogenetic relationships. The phylogenetic trees revealed the presence of seven HaNCED proteins, ten HaCCD proteins and four HaCCDL proteins. These results suggest that the HaNCED and HaCCD proteins within this subgroup may have functions similar to those of AtNCED and AtCCD proteins, respectively (Zhang et al. 2021). Similarly, the HaCCDL proteins may have functions similar to ClCCDLa, ClCCDLb, and CmCCDL proteins (Zhao et al. 2021).

Previous research suggests that positioning exons and introns within gene families is crucial to evolution (Yue et al. 2022). In this study, the analysis of gene structure and motifs indicated that members of the same population and clade had similar numbers and locations of exons, introns, and motifs, consistent with the topology of phylogenetic trees (Wei et al. 2022b). Exons and introns were present in all *CCO* genes. It is a characteristic of plants that the motifs of the *NCED* gene subfamily were discovered to be more conserved than those of the *CCD* subfamily. Furthermore, *cis*-regulatory elements play a crucial role in regulating gene expression at the transcriptional level, and these elements are located within the promoter region of genes. (Wei et al. 2022b).

A study of the *cis*-regulatory elements revealed that a significant proportion of *cis*-elements involved in the largest group, consisting of 213 (51.82%) elements, was responsive to light and contained motifs like Box 4, MRE, and G-box. The second-largest group, with 103 (25.06%) elements, was related to plant hormones and contained motifs like CGTCA-motif and TGACG-motif for MeJA response, TCA-element for SA response, GARE-motif, TATC-box, and P-box for GA response, ABRE for ABA response, and TGA-element for auxin response.

Drought and salinity stresses can reduce photosynthetic rates and transpiration in plants, resulting in crop yield losses. Stomata are crucial in plant photosynthetic activities and transpiration (Liang et al. 2011), (ALMAS et al. 2023). To investigate the potential functions of *HaCCO* genes, the expression patterns of *HaCCO* genes were studied using transcriptomic data from sunflower plants exposed to water deficit. The RNA-seq data analysis (GEO accession: GSE145709) identified *HaNCED16* and *HaNCED19* genes as potential candidates for developing drought stress-resistant sunflower varieties, while the *CCD* genes were not involved during drought stress. This suggests that these genes may be crucial in regulating sunflower physiology and development, specifically under drought stress conditions (Dhar et al. 2020). These genes might play a role in the production of ABA, a hormone that helps in the closure of stomata, reducing water loss through transpiration and improving water-use efficiency under drought-stress conditions (Bouvier et al. 2003).

Similarly, RNA-Seq data analysis for organ-specific gene expression (GEO accession: GSE221055) has provided valuable insights into potential pigmentation candidates within leaves. These identified genes are associated with functions related to chloroplasts and light response in plants (Zhu et al. 2010). The differential expression analysis of RNA-seq data revealed that the *HaCCD12* and *HaCCD20* genes had higher expression levels in leaves than in other organs, implying that they might play an important role in leaf pigmentation. This suggests that these genes may help synthesize and accumulate carotenoids in leaves, which leads to colouring of the tissues (Cárdenas-Conejo et al. 2023). They could regulate chlorophyll production, chloroplast biogenesis, or form photosynthetic pigment-protein complexes (Liang et al. 2020).

MicroRNAs (miRNAs) are crucial regulatory molecules in plants that play a significant role in several biological processes, including plant growth, development, and responses to biotic and abiotic stress (Y. Wang et al. 2023b), (Mazhar et al. 2023). They are highly conserved and exhibit specific functions. In our study, 16 (76.19%) *HaCCO* genes were found to have 60 miRNA target sites predicted based on previously described sunflower miRNAs. These findings suggest that miRNAs may play a role in the post-transcriptional regulation of *HaCCO* genes during sunflower development. This study’s outcomes provide fresh insights into the functional diversity and evolutionary dimensions of the *HaCCO* gene family. The extensive genome-wide identification and characterization performed in this study will help prepare sunflower varieties resistant to abiotic stresses.

## Conclusions

In this study, Twenty-one *CCO* genes were discovered in the genome of *H. annuus*. Based on structural analyses, the number of introns in *HaCCO* genes ranged from one to fourteen. The presence of cis-regulatory elements related to light responsiveness, development-related response, developmental and hormone responsiveness, and certain abiotic stress in the promoter of *HaCCO* genes suggested their function in the abiotic stress of sunflower. As identified by an RNA-seq data analysis, *HaNCED16* and *HaNCED19* genes can potentially be utilized to develop drought stress-resistant sunflower varieties for enhanced crop yield under water-limited conditions. The *HaCCD12* and *HaCCD20* genes were more active in leaves than in other organs, which suggests that they might play a role in leaf pigmentation. However, additional research, including gene cloning and functional analysis, is required to confirm the significance of these genes in various physiological and biological processes.

## Electronic supplementary material

Below is the link to the electronic supplementary material.

Supplementary Material 1

## Data Availability

All the data prepared during the research work is provided in the main body of the article or as supplementary materials. For publicly archived datasets, hyperlinks are provided in this manuscript to access already published data used in this article. Accession numbers of genetic materials used are also mentioned the article.

## Data Transparency

We have ensured that all data, materials, software applications, and custom code supporting the claims made in this article are in full compliance with field standards and provided in the article for the sake of reproducibility. It is important to note that we have taken into account the possibility of journal policies regarding research data sharing, considering the norms and expectations of our discipline.
