# Supplementary material for: Genome-wide identification and in-silico expression analysis of CCO gene family in sunflower (Helianthus annuus) against abiotic stress
Source: Plant Mol Biol. 2024 Apr 3;114(2):34. doi: 10.1007/s11103-024-01433-0 (PMC10991017; doi:10.1007/s11103-024-01433-0)
Supplement: Supplementary file 1 — Supplementary Material 1 [file 11103_2024_1433_MOESM1_ESM.docx]

**Supplementary Figures**


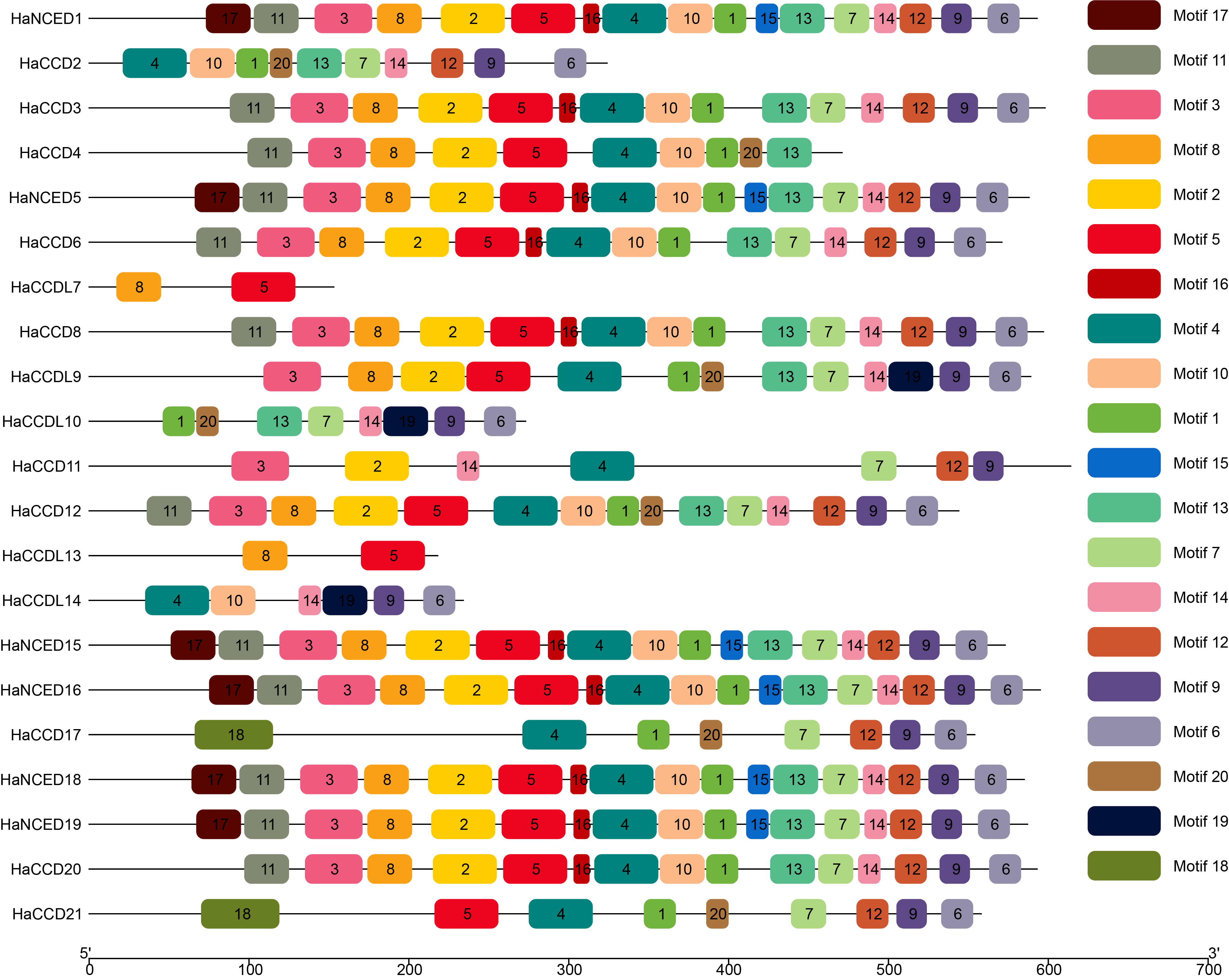


S Figure 1: Distributions of 20 motifs within sunflower CCO genes family


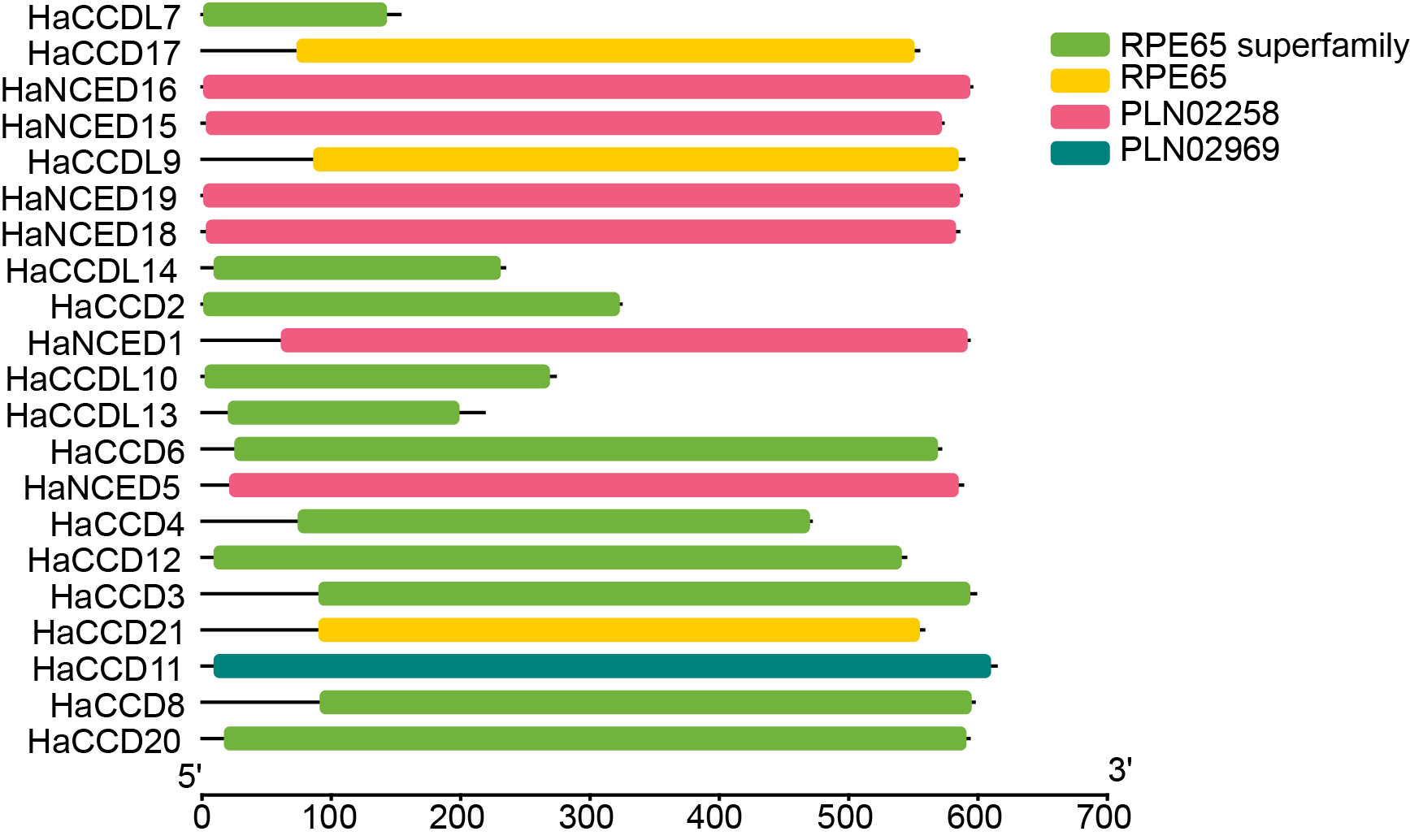


S Figure 2: The presence of RPE65 superfamily, RPE65, PLN02252, PLN02969 domains were present among all the HaCCO proteins


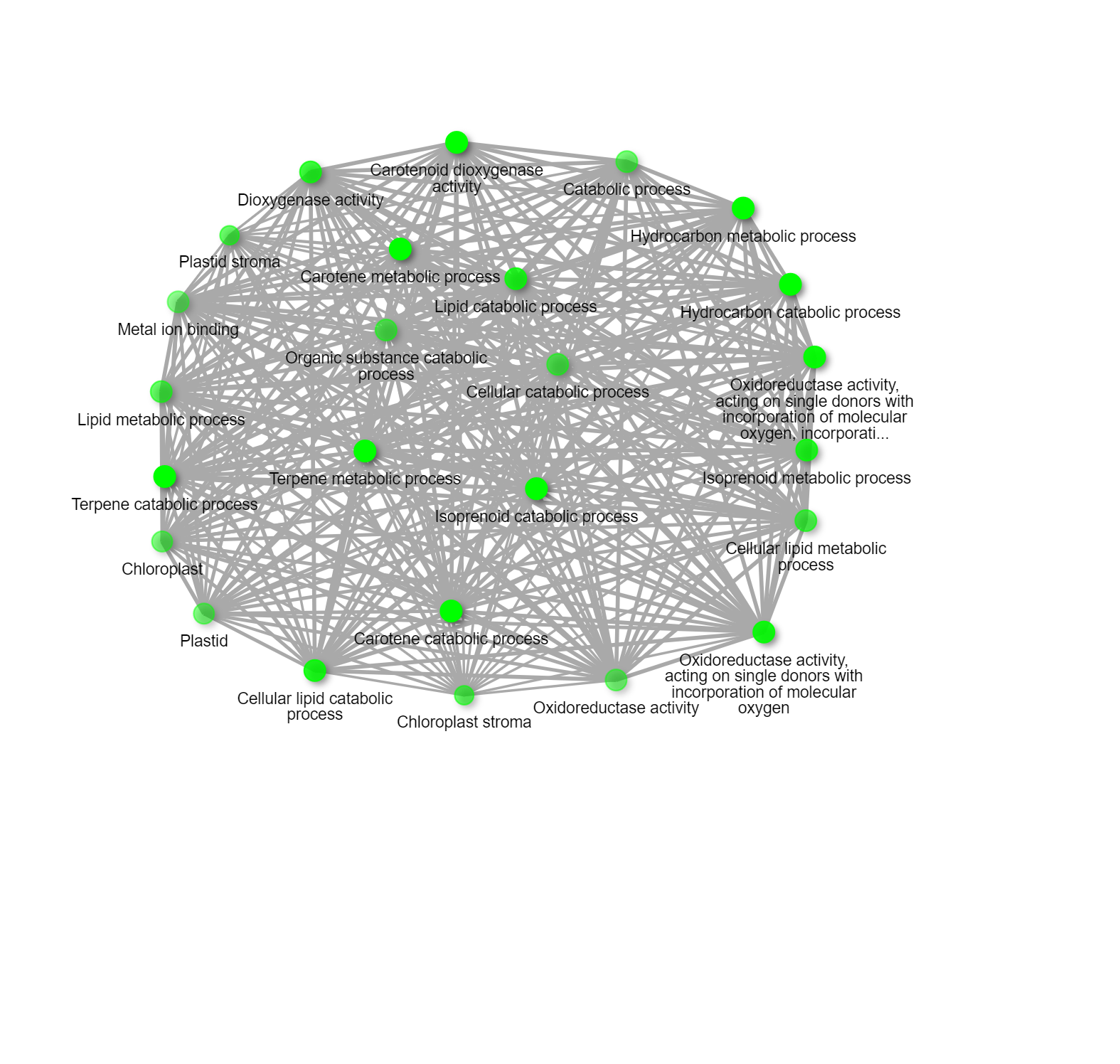


S Figure 3: Static Network Enrichment graph showing the network of HaCCO genes functions. Darker nodes are more significantly enriched gene sets. Bigger nodes represent larger gene sets. Thicker edges represent more overlapped genes

**Supplementary Tables**

**S Table 1: MiRNA Target Site Prediction and Validation**

| miRNA_Acc. | Target_Acc. | Expectation | UPE$ | miRNA_start | miRNA_end | Target_start | Target_end | miRNA_aligned_fragment | alignment | Target_aligned_fragment | Inhibition | | Target_Desc. | Multiplicity |
| --- | --- | --- | --- | --- | --- | --- | --- | --- | --- | --- | --- | --- | --- | --- |
| Han-miR395a | HaNCED18 | 4 | -1 | 1 | 21 | 906 | 926 | CUGAAGAGUUUGGGGGAACUU | :: ::..:::.:::::.: | ACCUUACCUUAAAUUCUUCGG | Cleavage | |  | 1 |
| Han-miRN5680 | HaCCD20 | 4 | -1 | 1 | 21 | 1597 | 1617 | CAUGUGCCCAUCUUCCCCAUC | :::: ::: ::::::.:..:: | GAUGAGGAUGAUGGGUAUGUG | Cleavage | |  | 1 |
| Han-miRN5713 | HaCCD20 | 4 | -1 | 1 | 21 | 1597 | 1617 | CAUGUGCCCAUCUUCCCCAUC | :::: ::: ::::::.:..:: | GAUGAGGAUGAUGGGUAUGUG | Cleavage | |  | 1 |
| Han-miRN5771 | HaNCED1 | 4 | -1 | 1 | 21 | 1026 | 1046 | ACAGCGUAAUUCUCCUUGGGC | : .:: :::::::: .:.::: | GAUCACGGAGAAUUUUGUUGU | Cleavage | |  | 1 |
| Han-miR1516c | HaNCED5 | 4.5 | -1 | 1 | 22 | 1038 | 1059 | AUGAAGCUUAAAAACACAUCGU | :: : ::: ::::::::::: | ACAAGUUGUGUUUAAGCUUCAA | Cleavage | |  | 1 |
| Han-miR160a | HaCCD20 | 4.5 | -1 | 1 | 21 | 1565 | 1585 | UGCCUGGCUCCCUGUAUGCCA | : :.: .:::::::::: :: | UUGUAGCUAGGGAGCCAGACA | Cleavage | |  | 1 |
| Han-miR160b | HaCCD20 | 4.5 | -1 | 1 | 21 | 1565 | 1585 | UGCCUGGCUCCCUGUAUGCCA | : :.: .:::::::::: :: | UUGUAGCUAGGGAGCCAGACA | Cleavage | |  | 1 |
| Han-miR160c | HaCCD20 | 4.5 | -1 | 1 | 21 | 1565 | 1585 | UGCCUGGCUCCCUGUAUGCCA | : :.: .:::::::::: :: | UUGUAGCUAGGGAGCCAGACA | Cleavage | |  | 1 |
| Han-miR160d | HaCCD20 | 4.5 | -1 | 1 | 21 | 1565 | 1585 | UGCCUGGCUCCCUGUAUGCCA | : :.: .:::::::::: :: | UUGUAGCUAGGGAGCCAGACA | Cleavage | |  | 1 |
| Han-miR162a | HaNCED19 | 4.5 | -1 | 1 | 21 | 657 | 677 | UCGAUAAACCUCUGCAUCCAG | : :::::.. .:::: ::::: | CGGGAUGUGCGGGUUGAUCGA | Cleavage | |  | 1 |
| Han-miR162b | HaNCED19 | 4.5 | -1 | 1 | 21 | 657 | 677 | UCGAUAAACCUCUGCAUCCAG | : :::::.. .:::: ::::: | CGGGAUGUGCGGGUUGAUCGA | Cleavage | |  | 1 |
| Han-miR169i | HaCCDL14 | 4.5 | -1 | 1 | 20 | 462 | 480 | UAGCCAAUGAUGACUUGCCU | : ::::: ::::::::.:: | UGUCAAGU-AUCAUUGGUUA | Cleavage | |  | 1 |
| Han-miR169k | HaCCDL14 | 4.5 | -1 | 1 | 20 | 462 | 480 | UAGCCAAUGAUGACUUGCCU | : ::::: ::::::::.:: | UGUCAAGU-AUCAUUGGUUA | Cleavage | |  | 1 |
| Han-miR390b | HaNCED18 | 4.5 | -1 | 1 | 21 | 379 | 399 | AAGCUCAGGAGGGAUAGCGCC | ::.:: :::::: .:::::: | GGUGCAAUCCCUGUUGAGCUA | Cleavage | |  | 1 |
| Han-miR390c | HaNCED18 | 4.5 | -1 | 1 | 21 | 379 | 399 | AAGCUCAGGAGGGAUAGCGCC | ::.:: :::::: .:::::: | GGUGCAAUCCCUGUUGAGCUA | Cleavage | |  | 1 |
| Han-miR390d | HaNCED18 | 4.5 | -1 | 1 | 21 | 379 | 399 | AAGCUCAGGAGGGAUAGCGCC | ::.:: :::::: .:::::: | GGUGCAAUCCCUGUUGAGCUA | Cleavage | |  | 1 |
| Han-miRN5689 | HaNCED1 | 4.5 | -1 | 1 | 21 | 672 | 692 | AUCGAGCUGCACAAUACACUG | : : ::::::..:.:.:.: | ACGCGGAUUGUGUGGUUUGGU | Cleavage | |  | 1 |
| Han-miRN5690 | HaNCED1 | 4.5 | -1 | 1 | 21 | 672 | 692 | AUCGAGCUGCACAAUACACUG | : : ::::::..:.:.:.: | ACGCGGAUUGUGUGGUUUGGU | Cleavage | |  | 1 |
| Han-miRN5692 | HaNCED1 | 4.5 | -1 | 1 | 21 | 672 | 692 | AUCGAGCUGCACAAUACACUG | : : ::::::..:.:.:.: | ACGCGGAUUGUGUGGUUUGGU | Cleavage | |  | 1 |
| Han-miRN5730 | HaCCD2 | 4.5 | -1 | 1 | 21 | 611 | 631 | UUGCGAUUUUUGUUUGUGCUU | : : :::::::.::.: :: | CUGGAAAAACAAAGAUUGAAA | Cleavage | |  | 1 |
| Han-miRN5732 | HaNCED16 | 4.5 | -1 | 1 | 21 | 1738 | 1758 | UUCGAGAGACGUUGUAUGAAG | .:::: . :::::.: ::::: | UUUCACGGAACGUUUAUCGAA | Cleavage | |  | 1 |
| Han-miRN5743 | HaNCED18 | 4.5 | -1 | 1 | 21 | 1362 | 1382 | CAAUUCAGAUCCGCCUCAAAA | : : :.: :::.:.:::::: | UAUCGGGUCGGGUUUGAAUUU | Cleavage | |  | 1 |
| Han-miRN5760 | HaNCED16 | 4.5 | -1 | 1 | 21 | 1738 | 1758 | UUCGAGAGACGUUGUAUGAAG | .:::: . :::::.: ::::: | UUUCACGGAACGUUUAUCGAA | Cleavage | |  | 1 |
| Han-miR159a | HaCCD6 | 5 | -1 | 1 | 21 | 548 | 568 | UUGGACUGAAGGGAGCUCCCU | : ::: ...:::::: ::: | GUGCAGCGUUUUUCAGUGCAA | Cleavage | |  | 1 |
| Han-miR159c | HaCCD6 | 5 | -1 | 1 | 21 | 548 | 568 | UUGGACUGAAGGGAGCUCCCU | : ::: ...:::::: ::: | GUGCAGCGUUUUUCAGUGCAA | Cleavage | |  | 1 |
| Han-miR159d | HaCCD6 | 5 | -1 | 1 | 21 | 548 | 568 | UUGGACUGAAGGGAGCUCCCU | : ::: ...:::::: ::: | GUGCAGCGUUUUUCAGUGCAA | Cleavage | |  | 1 |
| Han-miR159e | HaCCD6 | 5 | -1 | 1 | 21 | 548 | 568 | UUGGACUGAAGGGAGCUCCCU | : ::: ...:::::: ::: | GUGCAGCGUUUUUCAGUGCAA | Cleavage | |  | 1 |
| Han-miR159f | HaCCD6 | 5 | -1 | 1 | 21 | 548 | 568 | UUGGACUGAAGGGAGCUCCCU | : ::: ...:::::: ::: | GUGCAGCGUUUUUCAGUGCAA | Cleavage | |  | 1 |
| Han-miR159i | HaCCD6 | 5 | -1 | 1 | 21 | 548 | 568 | UUGGACUGAAGGGAGCUCCCU | : ::: ...:::::: ::: | GUGCAGCGUUUUUCAGUGCAA | Cleavage | |  | 1 |
| Han-miR159j | HaCCD6 | 5 | -1 | 1 | 21 | 548 | 568 | UUGGACUGAAGGGAGCUCCCU | : ::: ...:::::: ::: | GUGCAGCGUUUUUCAGUGCAA | Cleavage | |  | 1 |
| Han-miR159l | HaCCD6 | 5 | -1 | 1 | 22 | 548 | 569 | CUUGGACUGAAGGGAGCUCCCU | : ::: ...:::::: :::: | GUGCAGCGUUUUUCAGUGCAAG | Cleavage | |  | 1 |
| Han-miR162a | HaCCD4 | 5 | -1 | 1 | 21 | 276 | 296 | UCGAUAAACCUCUGCAUCCAG | .:::::: ::: :::::: : | UUGGAUGGAGAAGUUUAUAGU | Translation | | | 1 |
| Han-miR162b | HaCCD4 | 5 | -1 | 1 | 21 | 276 | 296 | UCGAUAAACCUCUGCAUCCAG | .:::::: ::: :::::: : | UUGGAUGGAGAAGUUUAUAGU | Translation | | | 1 |
| Han-miR170d | HaCCD21 | 5 | -1 | 1 | 21 | 580 | 600 | UGAUUGAGCCGUGCCAAUAUC | ::: ::::.. :.:::.:.: | AAUACUGGCGUUGUUCAGUUA | Translation | | | 1 |
| Han-miR170i | HaCCD21 | 5 | -1 | 1 | 21 | 580 | 600 | UGAUUGAGCCGUGCCAAUAUC | ::: ::::.. :.:::.:.: | AAUACUGGCGUUGUUCAGUUA | Translation | | | 1 |
| Han-miR170j | HaCCD21 | 5 | -1 | 1 | 21 | 580 | 600 | UGAUUGAGCCGUGCCAAUAUC | ::: ::::.. :.:::.:.: | AAUACUGGCGUUGUUCAGUUA | Translation | | | 1 |
| Han-miR396a | HaNCED16 | 5 | -1 | 1 | 21 | 247 | 267 | UUCCACAGCUUUCUUGAACUG | :::. ::::::.:: :: : | GUGUUUCAGAAAGUUGCGGCA | Cleavage |  | | 1 |
| Han-miR396b | HaNCED16 | 5 | -1 | 1 | 21 | 247 | 267 | UUCCACAGCUUUCUUGAACUG | :::. ::::::.:: :: : | GUGUUUCAGAAAGUUGCGGCA | Cleavage |  | | 1 |
| Han-miR396d | HaNCED16 | 5 | -1 | 1 | 21 | 247 | 267 | UUCCACAGCUUUCUUGAACUG | :::. ::::::.:: :: : | GUGUUUCAGAAAGUUGCGGCA | Cleavage |  | | 1 |
| Han-miR396e | HaNCED16 | 5 | -1 | 1 | 21 | 247 | 267 | UUCCACAGCUUUCUUGAACUG | :::. ::::::.:: :: : | GUGUUUCAGAAAGUUGCGGCA | Cleavage |  | | 1 |
| Han-miR396g | HaNCED16 | 5 | -1 | 1 | 21 | 247 | 267 | UUCCACAGCUUUCUUGAACUU | :::. ::::::.:: :: : | GUGUUUCAGAAAGUUGCGGCA | Cleavage |  | | 1 |
| Han-miR396h | HaNCED16 | 5 | -1 | 1 | 21 | 247 | 267 | UUCCACAGCUUUCUUGAACUU | :::. ::::::.:: :: : | GUGUUUCAGAAAGUUGCGGCA | Cleavage |  | | 1 |
| Han-miRN5680 | HaCCD3 | 5 | -1 | 1 | 21 | 1612 | 1632 | CAUGUGCCCAUCUUCCCCAUC | :: : ::: ::::::.:..:: | GAAGAGGAUGAUGGGUAUGUG | Cleavage |  | | 1 |
| Han-miRN5681 | HaCCD20 | 5 | -1 | 1 | 22 | 593 | 614 | UUAAAGCUUAGAAAAACGUCGU | :::: :::. :.: ::::: | CAAACGUGUUUUCAGGGUUUAA | Translation | | | 1 |
| Han-miRN5685 | HaNCED1 | 5 | -1 | 1 | 22 | 794 | 815 | UUUGGAUCAUCCGCUUUGGGGC | : ::.:: : :.::::...:: | UCACCGAAACCGGUGAUUUGAA | Cleavage |  | | 1 |
| Han-miRN5686 | HaNCED16 | 5 | -1 | 1 | 21 | 727 | 747 | CAAGUGGACUAAAAUGGCACC | :::. ::::::..:.:: | AACGCCGGUUUAGUUUAUUUU | Cleavage |  | | 1 |
| Han-miRN5701 | HaNCED16 | 5 | -1 | 1 | 21 | 727 | 747 | CAAGUGGACUAAAAUGGCACC | :::. ::::::..:.:: | AACGCCGGUUUAGUUUAUUUU | Cleavage |  | | 1 |
| Han-miRN5707 | HaNCED16 | 5 | -1 | 1 | 21 | 727 | 747 | CAAGUGGACUAAAAUGGCACC | :::. ::::::..:.:: | AACGCCGGUUUAGUUUAUUUU | Cleavage |  | | 1 |
| Han-miRN5713 | HaCCD3 | 5 | -1 | 1 | 21 | 1612 | 1632 | CAUGUGCCCAUCUUCCCCAUC | :: : ::: ::::::.:..:: | GAAGAGGAUGAUGGGUAUGUG | Cleavage |  | | 1 |
| Han-miRN5716 | HaCCDL13 | 5 | -1 | 1 | 21 | 405 | 425 | UUAAAUCUGAAUAAAUGGACC | :: :: :.:: ::::::.. | UUUCAAUGUGUUAAGAUUUGG | Cleavage |  | | 1 |
| Han-miRN5717 | HaCCDL13 | 5 | -1 | 1 | 21 | 405 | 425 | UUAAAUCUGAAUAAAUGGACC | :: :: :.:: ::::::.. | UUUCAAUGUGUUAAGAUUUGG | Cleavage |  | | 1 |
| Han-miRN5726 | HaCCDL13 | 5 | -1 | 1 | 21 | 405 | 425 | UUAAAUCUGAAUAAAUGGACC | :: :: :.:: ::::::.. | UUUCAAUGUGUUAAGAUUUGG | Cleavage |  | | 1 |
| Han-miRN5730 | HaCCD11 | 5 | -1 | 1 | 21 | 1389 | 1409 | UUGCGAUUUUUGUUUGUGCUU | ..:::.:..::::.:..: | GGAUGCAAGCGGAAAUUGUGA | Cleavage |  | | 1 |
| Han-miRN5731 | HaCCDL13 | 5 | -1 | 1 | 21 | 405 | 425 | UUAAAUCUGAAUAAAUGGACC | :: :: :.:: ::::::.. | UUUCAAUGUGUUAAGAUUUGG | Cleavage |  | | 1 |
| Han-miRN5733 | HaCCDL13 | 5 | -1 | 1 | 21 | 405 | 425 | UUAAAUCUGAAUAAAUGGACC | :: :: :.:: ::::::.. | UUUCAAUGUGUUAAGAUUUGG | Cleavage |  | | 1 |
| Han-miRN5742 | HaNCED15 | 5 | -1 | 1 | 22 | 1486 | 1507 | GCUCACUGCUCUAUCUGUCAUC | :: :: ::::: .: .:::::: | GAAGAGAGAUACGGGGGUGAGC | Translation | | | 1 |
| Han-miRN5771 | HaNCED16 | 5 | -1 | 1 | 21 | 1032 | 1052 | ACAGCGUAAUUCUCCUUGGGC | : .:: :::::::: ::. :: | GAUCACGGAGAAUUUCGUGGU | Cleavage |  | | 1 |
| Han-miRN5771 | HaNCED19 | 5 | -1 | 1 | 21 | 1008 | 1028 | ACAGCGUAAUUCUCCUUGGGC | : .:: :::::::: ::. :: | GAUCACGGAGAAUUUCGUGGU | Cleavage |  | | 1 |
| Han-miRN5771 | HaCCD12 | 5 | -1 | 1 | 21 | 822 | 842 | ACAGCGUAAUUCUCCUUGGGC | : ..: ::::::::.::: : | GAUUACCGAGAAUUAUGCUAU | Cleavage |  | | 1 |
| Han-miRN5774 | HaNCED16 | 5 | -1 | 1 | 22 | 809 | 830 | UUUGCAGUUUUCCAGUCACAUG | :: ::::.: :::::: ::... | CAGGUGAUUUGAAAACCGCGGG | Cleavage |  | | 1 |
